# The Defective Prophage Pool of *Escherichia coli* O157: Prophage–Prophage Interactions Potentiate Horizontal Transfer of Virulence Determinants

**DOI:** 10.1371/journal.ppat.1000408

**Published:** 2009-05-01

**Authors:** Md Asadulghani, Yoshitoshi Ogura, Tadasuke Ooka, Takehiko Itoh, Akira Sawaguchi, Atsushi Iguchi, Keisuke Nakayama, Tetsuya Hayashi

**Affiliations:** 1 Division of Bioenvironmental Sciences, Frontier Science Research Center, University of Miyazaki, Miyazaki, Japan; 2 Department of Veterinary Sciences, Faculty of Agriculture, University of Miyazaki, Miyazaki, Japan; 3 Division of Microbiology, Department of Infectious Disease, Faculty of Medicine, University of Miyazaki, Miyazaki, Japan; 4 Advanced Sciences Innovation Group, Mitsubishi Research Institute, Inc., Tokyo, Japan; 5 Bio-Frontier Research Center, Tokyo Institute of Technology, Kanagawa, Japan; 6 Department of Anatomy, Ultrastructural Cell Biology, Faculty of Medicine, University of Miyazaki, Miyazaki, Japan; University of Arizona, Tucson, United States of America

## Abstract

Bacteriophages are major genetic factors promoting horizontal gene transfer (HGT) between bacteria. Their roles in dynamic bacterial genome evolution have been increasingly highlighted by the fact that many sequenced bacterial genomes contain multiple prophages carrying a wide range of genes. Enterohemorrhagic *Escherichia coli* O157 is the most striking case. A sequenced strain (O157 Sakai) possesses 18 prophages (Sp1–Sp18) that encode numerous genes related to O157 virulence, including those for two potent cytotoxins, Shiga toxins (Stx) 1 and 2. However, most of these prophages appeared to contain multiple genetic defects. To understand whether these defective prophages have the potential to act as mobile genetic elements to spread virulence determinants, we looked closely at the Sp1–Sp18 sequences, defined the genetic defects of each Sp, and then systematically analyzed all Sps for their biological activities. We show that many of the defective prophages, including the Stx1 phage, are inducible and released from O157 cells as particulate DNA. In fact, some prophages can even be transferred to other *E. coli* strains. We also show that new Stx1 phages are generated by recombination between the Stx1 and Stx2 phage genomes. The results indicate that these defective prophages are not simply genetic remnants generated in the course of O157 evolution, but rather genetic elements with a high potential for disseminating virulence-related genes and other genetic traits to other bacteria. We speculate that recombination and various other types of inter-prophage interactions in the O157 prophage pool potentiate such activities. Our data provide new insights into the potential activities of the defective prophages embedded in bacterial genomes and lead to the formulation of a novel concept of inter-prophage interactions in defective prophage communities.

## Author Summary

Bacterial viruses, known as bacteriophages or phages, are major factors promoting horizontal gene transfer (HGT) between bacteria, and this activity has sparked new interest in light of the discovery that many sequenced bacterial genomes harbor multiple prophages carrying a wide range of genes, including those related to virulence. However, prophages identified from genome sequences often contain various genetic defects, and they have therefore been regarded as merely genetic vestiges, with no attention paid to their potential activities as mobile genetic elements. Enterohemorraghic *Escherichia coli* O157, which harbors as many as 18 prophages, is the most striking such example. The O157 prophages carry numerous genes related to O157 virulence, but most possess multiple genetic defects. In this study, we analyze the functionalities of O157 prophages and report that many of the apparently defective prophages are inducible and released from the O157 cells as particulate DNA and that some can be transferred to other *E. coli* strains. We should therefore regard these prophages as having high potential to disseminate virulence determinants. Our results further suggest that their activities as mobile genetic elements are potentiated by various types of interactions among the prophages, formulating a novel concept of inter-prophage interactions in defective prophage communities.

## Introduction

Horizontal gene transfer (HGT) is a major mechanism involved in bacterial evolution. In HGT between bacteria, viruses known as bacteriophages (or phages) play particularly important roles as gene transfer vehicles [1],[2]. Incoming temperate bacteriophages parasitize their hosts by integrating their genomes into the host genetic material. The additional genetic information that they provide to the host bacterium encodes various novel abilities, such as niche adaptation and the production of new virulence factors [2],[3]. Although phage-mediated HGT was first described in the 1950s in the conversion of *Corynebacterium diphtheriae* strains that did not produce a toxin to strains that did [4], studies in recent decades have identified a number of virulence determinants carried by phages [1]–[3], [5]–[7]. Furthermore, because numerous bacterial genomes have been sequenced, it has become increasingly clear that many bacterial genomes contain multiple prophages carrying a variety of genes [8]. However, the prophages identified from the genome sequences often contain genetic defects, such as deletions or disruptions of genes required for phage induction and propagation. Thus, such prophages are regarded simply as genetic remnants, and investigators tend to ignore the possibility that they might function as mobile genetic elements or participate in HGT.

Enterohemorrhagic *Escherichia coli* (EHEC) comprise a distinct class of *E. coli* strains that cause diarrhea, hemorrhagic colitis, and hemolytic uremic syndromes [9]. Among the various EHEC strains, the most dominant are the strains of serotype O157:H7 [10]. The genome of EHEC O157:H7 strain Sakai (referred to as O157 Sakai) contains 18 prophages (Sp1 to Sp18) and 6 prophage-like elements (SpLE1 to SpLE6), amounting to 16% of the total genome [11],[12]. These Sps and SpLEs have carried many virulence-related genes into the O157 Sakai genome, including the Shiga toxin genes (*stx1* and *stx2*), a set of genes for a type III secretion system (T3SS), numerous T3SS effector proteins, and transcriptional regulators for T3SS gene expression [11],[13]. A recent genomic comparison of O157 strains has further revealed that variation in prophage regions is a major factor generating the genomic diversity among O157 strains [14],[15].

An initial analysis indicated that, among the 18 Sps, 11 (Sp3–Sp6, Sp8–Sp12, Sp14 and Sp15) retain features of lambdoid phages, one (Sp13) has features similar to those of P2, one (Sp1) contains P4 features, and one (Sp18) retains Mu features. The other four Sps (Sp1 Sp7, Sp16, and Sp17) were unable to be assigned to particular phage families due to their chimeric or highly disrupted genomic backbones. Most of the lambdoid prophages resemble one another and contain various genetic defects ranging from frame-shift mutations to deletions and insertions of so-called insertion sequence (IS) elements [12]. Thus, a functional analysis of the prophage pool of O157 Sakai could reveal whether defective prophages have any biological activity and, perhaps more importantly, whether they have the potential to disseminate virulence factors among bacteria.

In the present study, we used bioinformatic analyses to re-evaluate the genomic structures of each Sp and to define their genetic defects by comparing them with their respective well-characterized prototype phages. We then systematically analyzed each Sp for its ability to excise itself from the host genome, replicate, and package its phage DNA. Our results indicate that many of the apparently defective prophages can excise themselves, replicate, and be released from O157 cells as particulate DNA. Furthermore, using Sp derivatives carrying a chloramphenicol-resistance (Cm^R^) gene, we demonstrated the transferability of apparently defective prophages to other *E. coli* strains. Our data indicate that defective prophages in the O157 prophage pool are not simply genetic remnants but have significant potential to act as mobile genetic elements that can mediate the spread of virulence-related genes from O157 to other bacteria. The results further suggest that various inter-prophage interactions in the prophage pool potentiate the biological activities of the defective prophages.

## Results

### *In silico* re-evaluation of the induction potential of Sps

**Figure 1:**
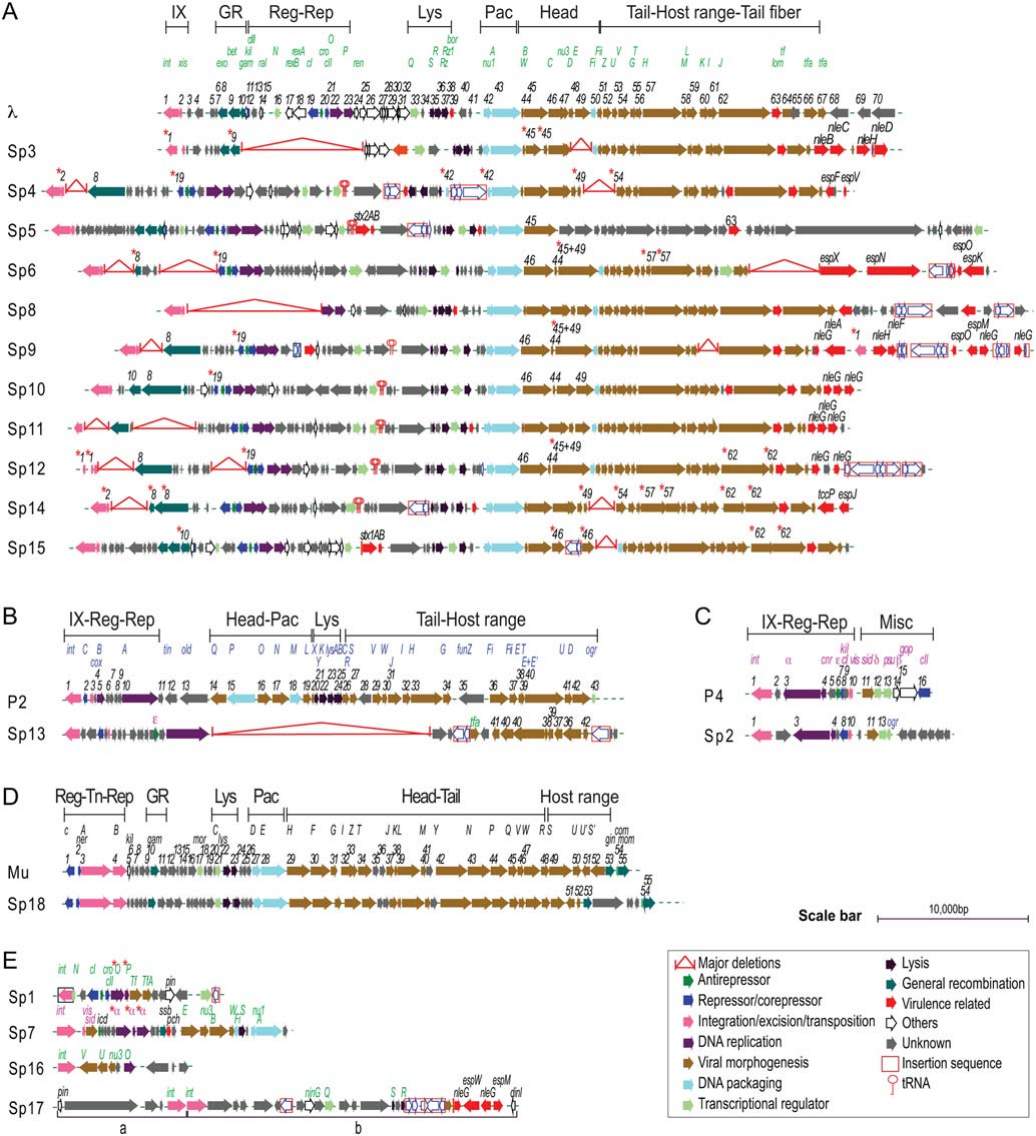
Alignment of 18 Sakai prophages (Sps) with the corresponding prototype phage genomes. Genomic organization of lambdoid Sps (A), P2-like Sp (B), P4-like Sp (C), and Mu-like Sp (D) are shown with corresponding prototype phage genomes. Genomic organization of other Sps are shown in (E). Genes on the prototypes are serially numbered from left to right according to their orf numbers [18],[29],[35],[36]. Only the regions in Sps showing variation in gene organization compared with the prototype genomes are numbered according to the prototype gene. Gene names on the prototypes are shown in green (λ), blue (P2), purple (P4), and black (Mu). Asterisks indicate the genes that are predicted to be non-functional. Phage genomic regions involved in integration/excision (IX), transposition (Tn), regulation (Reg), replication (Rep), general recombination (GR), packaging (Pac), cell lysis (Lys), morphogenesis (Portal, Head, Tail, and Host range) and miscellaneous (Misc) functions are indicated in each prototype genome. An enlarged version with more detailed information is presented in Figure S1.

The results of a genomic comparison of 18 Sps with their corresponding prototype phages are summarized below (Figure 1 and Table 1; see Figure S1 for more detail).

**Table 1:** Summary of the predicted essential phage functions in Sakai prophages.

Sakai Prophages	Repressor	Antirepressor	Integration/ Transposition^1^	Excision	Replication	General Recombination Function	Morphogenesis					DNA Packaging	Lysis
							Portal	Head	Major Tail	Minor Tail	Host Specificity		
Possessing conserved lambdoid features													
Sp3	−	−	D	+	−	D	D	−	+	+	+	+	+
Sp4	D	+	+	D	+	D	+	D	D	+	+	D	+
Sp5	+	+	+	+	+	+	+	U	U	U	U	+	+
Sp6	D	+	+	+	+	D	D	D	+	D	−	+	+
Sp8	−	−	+	+	+	−	+	+	+	+	+	+	+
Sp9	D	+	+	+	+	D	D	D	+	+	+	+	+
Sp10	D	+	+	+	+	+	+	+	+	+	+	+	+
Sp11	D	+	D	−	+	D	+	+	+	+	+	+	+
Sp12	D	+	D	+	+	D	D	D	+	+	D	+	+
Sp14	+	+	+	D	+	D	+	D	D	D	D	+	+
Sp15	+	+	+	+	+	D	+	−	+	+	D	+	+
P2- like prophage													
Sp13	+	+	+	+	(+)	NA	−	−	−	D	−	−	−
P4- like prophage													
Sp2	+	−	+	+	+	NA	NA^2^	NA^2^	NA^2^	NA^2^	NA^2^	NA^2^	NA^2^
Mu- like prophage													
Sp18	+	NA	+	NA	+	+	+	+	+	+	(+)	+	+
Other													
Sp1	+	+	D	−	D	−	−	−	−	−	−	−	−
Sp7	−	−	+	+	D	−	+	+	−	−	−	+	D
Sp16	−	−	+	−	D	−	−	−	+	−	−	−	−
Sp17	−	−	+	−	−	−	−	−	−	−	−	−	D

**+**, conserved; **(+)**, atypical; **D**, defective; **U**, cannot be predicted; **−**, absent; **NA**, not applicable.

1 Applicable only for the Mu-like prophage.

2 The morphogenesis and lysis functions of P4 are provided by the helper phage P2. The only morphogenesis function encoded on the P4 genome is the Sid, which determines the capsid size (Figure 1).

i) Lambdoid prophages: Among the 11 prophages with well-conserved lambdoid features, Sp3 and Sp8 lack repressor and anti-repressor functions (CI and Cro), which are at the center of the regulation of lysogenization and induction of lambdoid phages [16]–[19]. In the other lambdoid prophages, the repressors of Sp11 and Sp12 have been disrupted and those of Sp4, Sp6, Sp9, and Sp10 lack a peptidase domain that is required for SOS-induction (Figure 1A and Figure S2).

Integrase (Int), which mediates a bidirectional process of phage genome integration and excision [20], has been disrupted in Sp3, Sp11, and Sp12 (Figure 1A). Although Sp4 and Sp14 appear to encode intact integrases, their putative excisionase (Xis) proteins that regulate the directionality of the Int function [21] lack DNA binding motifs (data not shown) and are probably non-functional.

Replication initiator protein O and elongation protein P [22]–[24] are apparently functional in all lambdoid prophages except for Sp3 (Figure 1A). Whereas six (Sp6, Sp8, Sp9, Sp11, Sp14 and Sp15) have λ-type helicase loaders, three (Sp4, Sp10 and Sp12) have DnaC-type helicase loaders and Sp5 has an elongation protein almost identical to that of phage HK022, which belongs to the DnaB family (Figure S3).

Three Sps (Sp3, Sp5, and Sp15) have a general recombination system similar to that of phage λ, consisting of Exo, Bet, and Gam proteins (Figure 1A), but those of Sp3 and Sp15 have been disrupted. Seven Sps (Sp4, Sp6, Sp9–Sp12, and Sp14) possess a different type of recombination system that contains enterobacterial exodeoxyribonuclease VIII (Exo VIII)-type proteins instead of the λ-type exonuclease, but this system is intact only in Sp10.

All lambdoid Sps, except for Sp4, encode intact terminase, the key enzyme for DNA packaging [25],[26]. In Sp4, the *nu1* gene for the terminase small subunit protein has been disrupted by an IS insertion (Figure 1A).

The morphogenesis regions of Sp3, Sp4, Sp8, Sp11, Sp14, and Sp15 follow the gene organization of λ [18],[26], but Sp6, Sp9, Sp10, and Sp12 exhibit slightly different gene organizations in the head formation region (Figure 1A). Of these 10 Sps, all genes for morphogenetic function are conserved only in three (Sp8, Sp10, and Sp11). Sp15 (Stx1 prophage) also contains multiple defects in the morphogenic functions. Most of the putative morphogenic genes of Sp5 (Stx2 prophage) differ from those of λ and remain uncharacterized. However, another O157 Stx2-converting phage, called 933W, contains a set of genes nearly identical to that of Sp5 and has been shown to be fully active [27].

All 11 lambdoid Sps encode the Q protein, a regulator of late transcription. A full set of genes for cell lysis is also present in all lambdoid Sps, although some variation in gene organization is observed (Figure 1A). In Sp5, an IS-insertion has occurred upstream of the lysis region, but it has not disrupted any protein-coding genes; indeed, an Sp5 derivative has been shown to transfer from O157 Sakai to K-12 [28].

Based on our *in silico* analysis of the 11 lambdoid Sps, we predicted that (1) three (Sp3, Sp11, and Sp12) would no longer be able to excise themselves from the host chromosome and (2) the other eight prophages would be excisable, but seven (Sp4, Sp6, Sp8–Sp10, Sp14, and Sp15) would have some defects in morphogenesis or other functions, and only Sp5 would be capable of the full complement of viral functions (Table 1).

ii) P2 and P4-like prophages: Phage P2 functions as a helper for the satellite phage P4 [29]–[33]. The P2–P4 couple in O157 Sakai is atypical because homologues of the P2 *ogr* gene and the P4 *ε* gene [30],[31] are present in Sp2 (P4-like phage) and Sp13 (P2-like phage), respectively (Figure 1B and 1C). In addition, Sp13 lacks most phage functions, including most of the morphogenetic genes (Figure 1B and Table 1); thus, it may no longer propagate by itself or work as a helper for Sp2.

iii) Mu-like prophage: Sp18 is predicted to be intact and spontaneously inducible like the prototype Mu phage because most features for Mu-like phages [34],[35] are conserved (Figure 1D). Sp18 also contains an invertible host-specificity region, but the encoded genes are distinct from those of Mu [35].

iv) Others: The other four prophage genomes have been severely degraded, but all may have been derived from lambdoid phages because many of their residual genes are homologous to λ genes (Figure 1E). Reassessment of the Sp17 region revealed that two prophages (referred to as Sp17a and Sp17b) have been integrated in tandem in this region. Sp7 shows interesting chimeric features of lambdoid and P4-like phages. Sp7 encodes a P4-like Int, as well as a P4-like Xis (Vis-homologue) [36]. In addition, Sp7 contains a gene similar to the P4 *α* gene, but the gene has been disrupted by multiple frame-shift mutations.

### DNA amplification and transcriptional response of the prophage pool during induction

**Figure 2:**
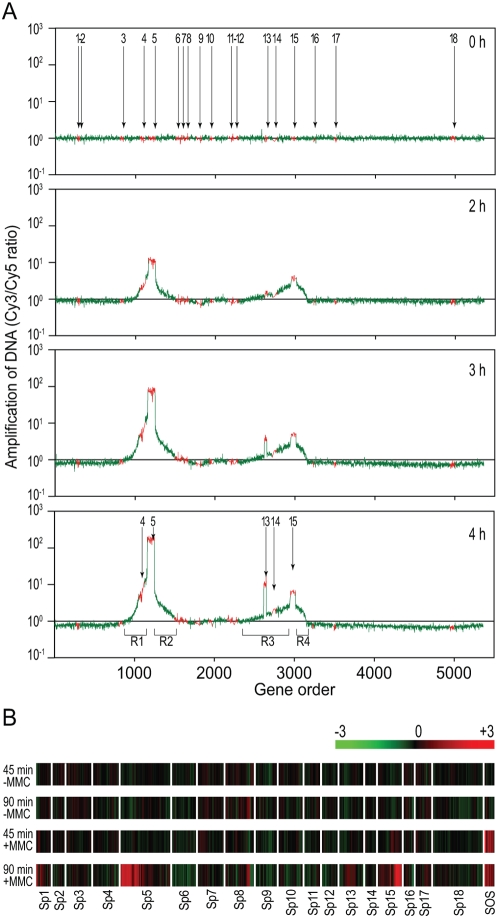
Microarray analysis of the DNA amplification and transcriptional changes of prophages induced by MMC treatment. (A) Prophage DNA amplification induced by MMC. Total cellular DNA was prepared from O157 Sakai cells treated with MMC and amplification of prophage regions was analyzed using the oligo DNA microarray. Genes on the O157 Sakai chromosome are shown on the x-axes according to their genomic context. The y-axes indicate the ratios of hybridization signals of test DNA preparations relative to that from the reference DNA for the 0-h sample. The test DNA was prepared from the O157 Sakai cells collected at the indicated time points after MMC was added to the culture. The data from the probes representing prophage and backbone genes on the O157 Sakai chromosome are shown in red and green, respectively. All prophage regions (Sp1–Sp18) on the O157 Sakai chromosome are indicated in the 0-h plot, and selectively enriched prophage regions are indicated in the 4-h plot. Chromosome regions amplified by the regional replication of Sp5 and Sp15 (R1 and R2 by Sp5, R3 and R4 by Sp15) are also indicated in the 4-h plot. (B) Transcriptional changes of the prophage genes induced by MMC. The color bar indicates relative expression levels. Total RNA was prepared from the cells collected at the 45-min and the 90-min time points after the addition of MMC. RNA prepared from the cells collected at the 0-min point was used as the reference. RNA prepared from cells left untreated with MMC was also analyzed with the same protocol (−MMC). Only the data for genes in the 18 prophage regions (Sp1–Sp18) are shown according to their genomic context, along with those for the 10 chromosome genes (*sulA*, *dinI*, *hlyE*, *umuD*, *umuC*, *recX*, *recA*, *dinD*, *lexA*, and *dinF*) that are known to be induced by MMC treatment (SOS) [39],[40]. Average values obtained from two independent experiments are shown. Note that specific probes were unable to be designed for many genes on lambdoid phages because they have nearly identical sequences. These genes were excluded from this analysis.

To experimentally evaluate the inducibility of each Sp, we first examined the amplification of prophage DNA upon mitomycin C (MMC) treatment of O157 Sakai cells using an oligo DNA microarray. Cell lysis started 2 to 3 hr after the addition of MMC (1 µg/ml) to the early log-phase culture, and the optical density (OD) returned to basal levels within 6 to 8 hr as a result of cell lysis (Figure S4). We isolated total cellular DNA from aliquots of cultures at 1-hr intervals from 0 hr to 4 hr after the addition of MMC. We then analyzed the total DNA using the microarray (Figure 2A).

We observed selective amplification of Sp5, Sp13 and Sp15 regions, although amplification of the Sp13 regions was delayed relative to that of the other two regions. Interestingly, the Sp5-flanking regions exhibited significant amplification (R1 and R2 in Figure 2A). The Sp15-flanking regions also showed substantial amplification (R3 and R4 in Figure 2A), but the amplification of the R3 and R4 regions was asymmetric and smaller than that for the Sp5-flanking regions. A similar phenomenon in phage λ is known as the “regional replication” of prophage-flanking regions. In “regional replication,” upon induction, the chromosomal regions flanking the λ prophage genome replicate together with the λ genome that remains to be excised from the chromosome [37]. Similar phenomena have also been reported for other lambdoid phages [38]. Importantly, the amplified prophage-flanking regions of Sp5 and Sp15 included prophages Sp4 and Sp14, respectively (Figure 2A). Our preliminary microarray analysis of a spontaneous Sp5 deletion mutant confirmed that the amplification of the Sp5-flanking region in response to MMC treatment requires the presence of Sp5 (data not shown).

We also analyzed transcriptional changes of the prophage genes upon MMC treatment using the microarray (Figure 2B). A large number of chromosomal genes were up- or down-regulated by MMC treatment (data not shown), as described in K-12 [39],[40]. Among the prophage genes, we observed a marked increase in the transcript levels of the Sp5, Sp13, and Sp15 genes, especially in their early regions, which is in agreement with their selective DNA amplification upon MMC treatment (Figure 2A). No significant transcriptional changes in response to MMC treatment were detected for most genes of other prophages, except for those of Sp1. Although this highly degraded lambdoid prophage showed no DNA amplification upon MMC treatment (Figure 2A), many of its residual early genes exhibited clear induction. The biological significance of this phenomenon is unknown.

### Excision, circularization, and replication of prophage genomes

**Figure 3:**
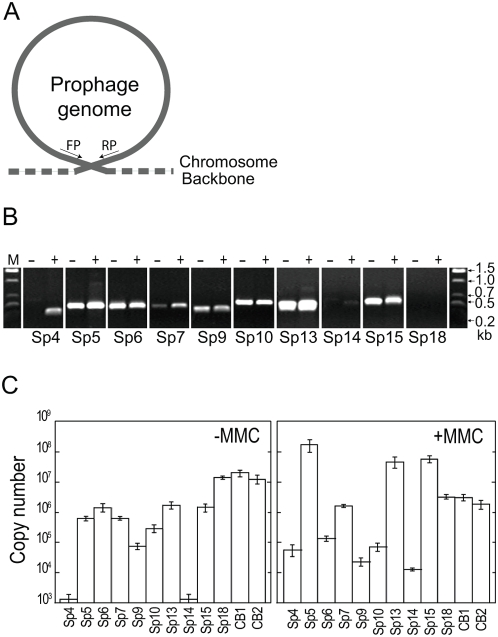
Excision, circularization, and replication of prophages. (A) Schematic representation of the strategy to detect excised and circularized prophage genomes by PCR. (B) Detection of PCR products derived from excised and circularized prophage genomes by agarose gel electrophoresis. Total cellular DNA isolated from untreated (−) and MMC-treated cells (+) was analyzed. We examined all Sps, but only the data from the nine prophages that gave positive results are shown. The data of Sp18, a Mu-like phage whose DNA is not circularized, are shown as a negative control. (C) Quantification of circularized phage genomes in the total cellular DNA isolated from untreated (−MMC) and MMC-treated cells (+MMC) using quantitative PCR (qPCR). The data were obtained from three independent analyses, and average copy numbers for each prophage genome are shown. Bars indicate standard deviations. The Sp18 DNA and the chromosomal DNA from two chromosome regions (CB1 and CB2) were quantified as controls.

To determine whether the Sps amplified by MMC treatment are excised from the host chromosome into a circular form and whether other Sps are excisable but to a much lesser extent, we looked for the presence of circular forms of all Sps using PCR amplification of the attachment site (*attP*)-flanking regions that are generated by excision and circularization (Figure 3). In MMC-treated O157 Sakai cells, we detected circularized DNA not only from Sp5, Sp13, and Sp15 but also from Sp6, Sp7, Sp9, and Sp10 (Figure 3B). In addition, the circular forms of Sp4 and Sp14 genomes were detected, although the amount of circularized Sp14 was significantly lower than those of the other prophages. Furthermore, we also detected the circular form of DNA for all of these prophages, except for Sp4 and Sp14, in O157 Sakai cells that had not been treated with MMC. These results indicate that the nine prophages can be excised into a circular form by MMC-mediated or spontaneous induction and that the Sp4 and Sp14 genomes, which were amplified by regional replication, can also be excised and cyclized. Circularized Sp18 DNA was not detected, which is consistent with previous results for the prototype Mu phage [34],[35].

By sequencing the PCR products obtained in this analysis, we confirmed that the circularized prophage genomes arose by site-specific recombination between the left and right phage attachment sites (*attL* and *attR*). This analysis also allowed us to precisely determine the core attachment sequences of these nine prophages (Figure S5). The results largely agreed with our previous predictions, except for the results with Sp9 [12]. The *attL* and *attR* sites of Sp9 are each located 121 bp upstream of the predicted positions, and we identified the true core sequence of 28 bp.

We also used the same strategy to analyze six prophage-like elements (SpLEs) of O157 Sakai. However, we detected no excised and circularized DNA from any of these elements, either in untreated cells or in cells treated with MMC (data not shown). This suggests that these elements have no (or very low) mobility or require other types of stimuli to be mobilized.

### Quantification of prophage replication

Because we found that many Sps are excised into circular forms, we quantified the circularization and/or replication of these Sps using quantitative PCR (qPCR) (Figure 3). In untreated cells, we detected similar amounts of circularized DNA of Sp5, Sp6, Sp7, Sp13, and Sp15 and a slightly lower level of circularized Sp9 DNA. In cells treated with MMC, the relative amounts of circularized DNA of Sp5, Sp13, and Sp15 increased to approximately 300, 30, and 40 times higher than their levels in untreated cells, respectively (Figure 3C). In contrast, the levels of Sp6, Sp9 and Sp10 were lower than in the untreated cells. It is noteworthy that, in complete agreement with the results of the qualitative PCR analysis (Figure 3B), considerable amounts of circularized Sp4 and Sp14 were generated in the MMC-treated cells, whereas hardly any was detected in the untreated cells. Sp18 also proved to be non-inducible by MMC, as described for phage Mu [35].

DNA microarrays were used to monitor prophage induction [38],[41]. Microarray analysis can provide a gross image of the replication pattern of prophages augmented by MMC treatment, as seen in Figure 2, but it cannot detect spontaneous induction of prophages. Thus, as our present data show (Figure 3, see also Figure S6), qPCR analysis is required to obtain the true picture of prophage induction. This may also be true in transcriptome analysis of prophage genes (Figure 2B).

In general, the stability of a prophage is tightly coupled with the physiology of the host cell. Under conditions that generate DNA injury—in the present study through MMC treatment—prophages are de-repressed by a RecA-mediated mechanism (the SOS response) to enter the lytic pathway [42]. The RecA protein stimulates self-cleavage of the repressor protein, which leads to the expression of genes required for the lytic pathway [43],[44]. The non-inducible nature of Sp6, Sp9 and Sp10 by MMC treatment is consistent with the fact that the peptidase motif is missing in the repressors of these lambdoid Sps (Figure S2). Phage P2 is insensitive to the SOS response and is thus non-inducible by MMC treatment, because its repressor intrinsically lacks the peptidase motif [30]. The repressor of Sp13, a P2-like prophage, also lacks the peptidase motif. Thus, the MMC-mediated induction of Sp13 observed in this analysis is remarkable (Figures 2, Figure 3, and Figure S6). Although the mechanism is yet to be elucidated, a P4 *ε*-like gene encoded on Sp13 (Figure 1 and Figure S2) may be involved in this unique behavior because the P4 *ε* gene product de-represses the P2 genome by binding to the P2 repressor [30],[31].

### Packaging of prophage DNA

**Figure 4:**
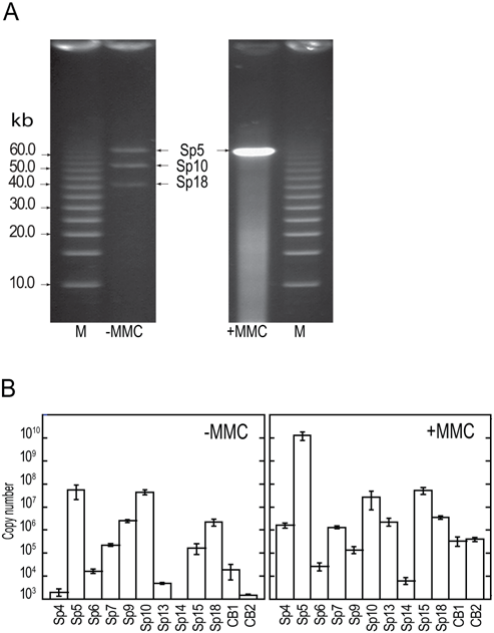
Phage DNA packaged into phage particles. (A) FIGE analysis of packaged phage DNA in untreated (−MMC) and MMC-treated (+MMC) cultures. Phage particles were collected from culture supernatants by PEG/NaCl precipitation. DNA preparations applied to the FIGE gel were obtained from 50-ml (untreated) and 1-ml (MMC-treated) cultures. (B) Quantification of packaged phage DNA in the culture supernatants obtained from untreated (−MMC) and MMC-treated (+MMC) O157 Sakai cells. DNase-resistant phage DNA was quantified by qPCR using the primers that were used in Figure 3. DNA preparations equivalent to the 1-ml culture supernatant were used as template DNA. The data were obtained from three independent analyses, and the average copy numbers for each prophage genome are shown. Bars indicate standard deviations. The Sp18 DNA and the chromosomal DNA from two chromosome regions (CB1 and CB2) were monitored as controls.

To investigate whether the Sps that were circularized and replicated by spontaneous or MMC-mediated induction could be packaged into phage particles, we first attempted field inversion gel electrophoresis (FIGE) analysis of DNA isolated from phage particles. Particles were taken from supernatants of bacterial cultures that were either treated with MMC or left untreated (Figure 4A). In the untreated sample, we detected packaged DNA of Sp5, Sp10, and Sp18. However, upon MMC treatment, a large amount of Sp5 DNA accumulated and generated extensive smearing that prevented the visualization of minor species of packaged phage DNA.

We therefore quantified particulate DNA of each Sp by qPCR using the same set of PCR primers used for the quantification of intracellular phage DNA (Figure 4B). In the untreated sample, we detected DNase-resistant forms of DNA for at least five prophages (Sp5, Sp7, Sp9, Sp10, and Sp15). They included two Sps (Sp9 and Sp15) that contain genetic defects in head formation (Figure 1 and Table 1). This result suggests that the defects of these two prophages were complemented, probably by other prophages that provided all or some of the gene products required for head formation. The amount of Sp6 DNA was marginal compared with the control chromosomal DNA (CB1 and CB2 in Figure 4B). The Sp13 DNA appears to be inefficiently packaged or unstable. As expected from the data on phage Mu, the Sp18 DNA was efficiently packaged.

In the MMC-treated sample, a large amount of packaged Sp5 DNA was detected, reaching levels of ≥10^10^ molecules per milliliter of culture. Although at much lower levels, we detected considerable amounts of packaged DNA from at least six other Sps (Sp4, Sp7, Sp10, Sp13, Sp15, and Sp18). Interestingly, this group includes Sp13, which also lacks the genes for head formation and DNA packaging (Figure 1 and Table 1). Of the two Sps (Sp4 and Sp14) whose genomes are amplified only by the regional replication of Sp5 and Sp15, respectively, we detected packaged Sp4 genomic DNA, although it also contains defects in head formation and DNA packaging. Thus, these defects of Sp13 and Sp4 must have also been complemented by other prophages.

### Infectivity/transferability of the particulate prophage DNA

**Figure 5:**
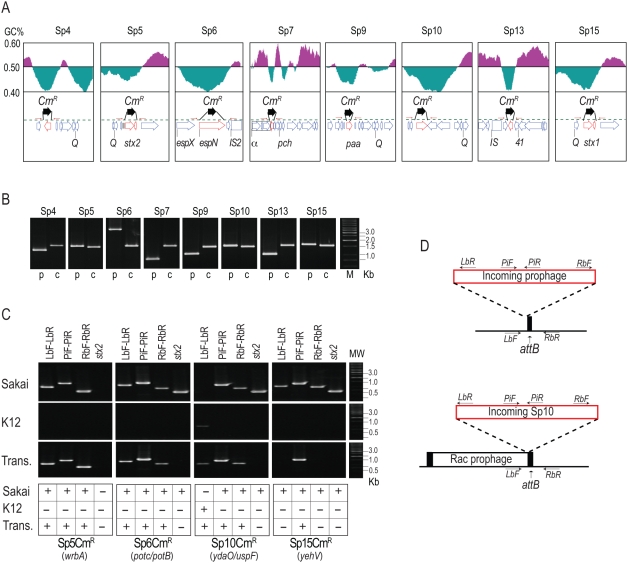
Construction of Cm^R^-marked prophages and analysis of their transfer and lysogenization into K-12 strains. (A) Strategy to construct Cm^R^-marked prophages. The gene organizations and GC contents of the regions flanking each target gene that was replaced by the Cm^R^ gene cassette are shown. (B) Verification of gene replacement using PCR. PCR products obtained from the O157 Sakai parent strain (p) and mutant strains carrying the Cm^R^ cassette in each prophage (c) are shown. (C,D) PCR examination of the transfer of Cm^R^-marked prophages from O157 Sakai to K-12. PCR products obtained from O157 Sakai (Sakai) and K-12 (K12) cells and the transductants (Trans) of Cm^R^-marked Sp5, Sp6, Sp10, and Sp15 are shown in (C). Absence of the *stx2* gene in all transductants was also confirmed. Integration sites of each prophage in O157 Sakai are indicated in parentheses. Integration patterns of each prophage in K-12 and positions of PCR primers used are shown in (D). The chromosomal attachment site (*attB*) for Sp10 was occupied by the Rac prophage, and incoming Sp10 was integrated into the K-12 chromosome using the *attR* site of the Rac prophage. In the Sp15-transductant, the Cm^R^-marked Sp15 was not found at the *yehV* locus, where Sp15 is integrated in O157 Sakai. Our preliminary result with the Sp15 transductants indicates that the Cm^R^ gene on Sp15 was transferred to K-12 by a chimeric phage generated by recombination between the Sp5 and the Cm^R^-marked Sp15 genomes (see text).

To examine the transferability of packaged Sp genomes, we marked the eight Sps (Sp4–Sp7, Sp9, Sp10, Sp13, and Sp15) by replacing “moron” genes of each phage genome, which are not required for phage propagation, with a Cm^R^ gene cassette (Figures 5A and 5B). The *stx2* gene in Sp5 and *stx1* gene in Sp15 were replaced with the cassette. Incorporation of the Cm^R^ cassette into the prophage genomes did not affect DNA packaging because the levels of particulate phage DNA detected for each Cm^R^-derivative were similar to those observed for the wild-type O157 Sakai (data not shown).

To examine whether the Cm^R^ marker can be transferred to two K-12 derivatives (strains MG1655 and MC1061), we analyzed the culture supernatants prepared from O157 Sakai containing each Cm^R^-marked Sp derivative with or without MMC treatment. We found that the Cm^R^ gene on four Sps (Sp5, Sp6, Sp10, and Sp15) is transferable to K-12 and stably maintained, although the efficiency of transfer was low in all cases except that of Sp5 (Table 2). Among the four Sps, three (Sp5, Sp6, and Sp10) were integrated at the same chromosomal loci in K-12, as in O157 Sakai (Figure 5C). The integration site of Sp10 was already occupied by the Rac prophage in K-12, but the Sp10 derivative was integrated in tandem with Rac using the *attR* sequence of Rac as the *attB* site (Figure 5D). In contrast, the Sp15 derivative was not integrated into the *yehV* locus in K-12, the chromosomal locus where Sp15 is present in O157 Sakai (Figure 5C). This suggests that recombination occurred between Sp15 derivatives and other Sps that allowed the transfer of the *Cm^R^* gene (by which the *stx1* gene was replaced) to K-12 (see the next section).

**Table 2:** Excision, replication, and packaging abilities of Sakai prophages and their transfer, lysogenization, and propagation in K-12.

Prophages	Circularization	Replication	Packaging	Transfer efficiency^a^				Lysogenization		Transfer from K-12 to K-12^b^
				MG1655		MC1061		MG1655	MC1061	
				−MMC	+MMC	−MMC	+MMC			
Sp1	−	−	−	N	N	N	N	N	N	N
Sp2	−	−	−	N	N	N	N	N	N	N
Sp3	−	−	−	N	N	N	N	N	N	N
Sp4	+	+	+	0	0	0	0	N	N	N
Sp5	+	+	+	6(4)	746(141)	2(1)	115(36)	+	+	+
Sp6	+	+	+	0	0	4(3)	0	N	+	−
Sp7	+	+	+	0	0	0	0	N	N	N
Sp8	−	−	−	N	N	N	N	N	N	N
Sp9	+	+	+	0	0	0	0	N	N	N
Sp10	+	+	+	0	0	47(16)	2	N	+	−
Sp11	−	−	−	N	N	N	N	N	N	N
Sp12	−	−	−	N	N	N	N	N	N	N
Sp13	+	+	+	0	0	0	0	N	N	N
Sp14	+	+	−	N	N	N	N	N	N	N
Sp15	+	+	+	0	8(5)	0	1	+	+	+
Sp16	−	−	−	N	N	N	N	N	N	N
Sp17	−	−	−	N	N	N	N	N	N	N
Sp18	N	+	+	N	N	N	N	N	N	N

a Transfer to K-12 was examined using Cm^R^–marked prophages. Average numbers of Cm^R^ colonies obtained in three or four independent experiments are shown. Standard deviations are given in parentheses. N; not applicable or not determined.

b Both K-12 strains MG1655 and MC1061 were used as recipients.

Among the three Sps that were successfully transferred to K-12, the Sp5 derivative produced infective phage particles in K-12 (Table 2). In contrast, we could not detect the production of infective Sp6 or Sp10 derivatives in K-12. This result suggests that these two Sps may require the support of other Sps, available only in the O157 cell, to produce infective phage particles efficiently.

### Generation of new Stx-transducing phages by recombination of Sp15 with Sp5

**Figure 6:**
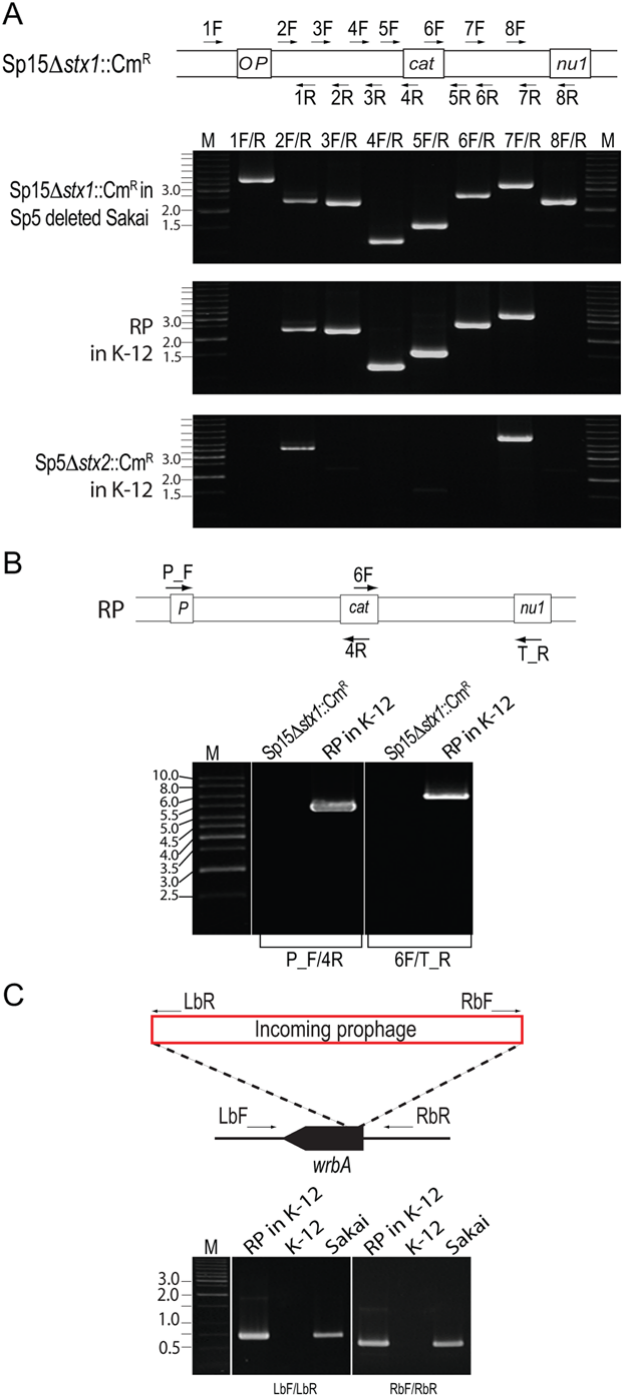
Generation of a chimeric phage by recombination between the Cm^R^-marked Sp15 derivative and Sp5. (A) PCR scanning analysis of the *stx1*-flanking region of a chimeric recombinant phage (RP) in a K-12 Sp15*Δstx1*::Cm^R^ transductant. Positions of the PCR primers on the Sp15*Δstx1*::Cm^R^ genome are schematically shown at the top. For comparison, the *stx1*-flanking region of Sp15*Δstx1*::Cm^R^ in the donor strain O157 Sakai and the corresponding *stx2*-flanking region of Sp5*Δstx2*::Cm^R^ in its K-12 transductant were analyzed using the same set of primers. In this analysis, a spontaneous Sp5-deletion mutant of the Sp15*Δstx1*::Cm^R^-containg O157 Sakai was used because Sp15 and Sp5 genomic regions between the *P* gene and the *stx1* (or *stx2*) gene and between the *stx1* (or *stx2*) gene and the *nu1* gene contain highly homologous sequences (see Figure S7 for more details). (B) PCR analysis of the chimeric phage. The *stx1*-flanking region of the Sp5/Sp15 recombinant phage in K-12 was analyzed using two primer pairs. The positions of the primers on the chimeric phage genome are schematically shown at the top. Primers P_F and T_R are specific to the *P* and *nu1* genes of Sp5, respectively. As a control, an O157 Sakai-derivative containing Sp15*Δstx1::Cm^R^* was analyzed. PCR products were obtained by the two primer pairs (4.9 kb and 6.4 kb in size, respectively) only from the K-12 derivative carrying the recombinant phage (RP in K-12). (C) PCR analysis of the *wrbA* locus of a K-12 derivative carrying the chimeric phage (RP in K-12), K-12, and O157 Sakai. Integration of an Sp5-like phage into the *wrbA* locus (the integration site of Sp5 in O157 Sakai) in the K-12 derivative was confirmed by PCR using two primer pairs. Positions of the primer are schematically shown at the top. Primers LbR and RbF are specific to the left and right ends of the Sp5 genome, respectively.

To analyze the Cm^R^-marked Sp15 (Sp15*Δstx1*::Cm^R^) transductants, we first performed PCR scanning analysis of the *stx1*-flanking region of an Sp15*Δstx1*::Cm^R^-transductant of K12 MG1655 (Figure 6A, see Figure S7 for more details). The results indicated that the transductant contains an Sp15*Δstx1*::Cm^R^-derived DNA segment covering the *stx1* region, but also that some recombination had occurred between the *P* and *stx1* (replaced by the Cm^R^ cassette) genes and between the *stx1* and *nu1* genes in the Sp15*Δstx1*::Cm^R^ genome.

By DNA sequence homology analysis between Sp15 and other Sps, we found that, although many lambdoid Sps contain one or more genomic segments that are highly homologous to the *stx1*-flanking region of Sp15, only Sp5 contains both segments homologous to the upstream and downstream regions of the *stx1* gene (Figure S7). These Sp5 segments are also present in the upstream and downstream regions of the *stx2* gene. This suggested that the Cm^R^ Sp15 derivative transferred to K-12 may have been generated by recombination between Sp15*Δstx1*::Cm^R^ and Sp5. We therefore analyzed the genome of the Cm^R^ Sp15 derivative by PCR using two primer pairs: those specific to the Cm^R^ cassette and the Sp5 *P* gene and those specific to the Cm^R^ cassette and the Sp5 *Nu1* gene (Figure 6B and Figure S7). The two primer pairs yielded 4.9-kb and 6.4 kb amplicons, respectively, both of which were absent in the donor O157 Sakai derivative containing Sp15*Δstx1*::Cm^R^. Furthermore, we confirmed that the Sp15*Δstx1*::Cm^R^ transductant contains an Sp5-like phage in the *wrbA* locus, the integration site of Sp5 (Figure 6C and Figure S7). All these data indicated that the Cm^R^ cassette-carrying phage is a chimeric phage that was generated by replacing the *stx2* regions of Sp5 with the *stx1* region of Sp15*Δstx1*::Cm^R^.

We analyzed 21 additional Sp15*Δstx1*::Cm^R^ transductants using the same methods. The results indicated that all of the transductants contain chimeric phages of Sp15*Δstx1*::Cm^R^ and Sp5 (data not shown). However, the data from our preliminary sequence analysis of the PCR products covering recombination points suggested that several (at least four) types of chimeric phages had been generated (more details of these chimeric phages will be described elsewhere). It may also be worth noting that this phenomenon was observed only in MMC-treated O157 cells (Table 2).

### Electron microscopic examination of phage particles

**Figure 7:**
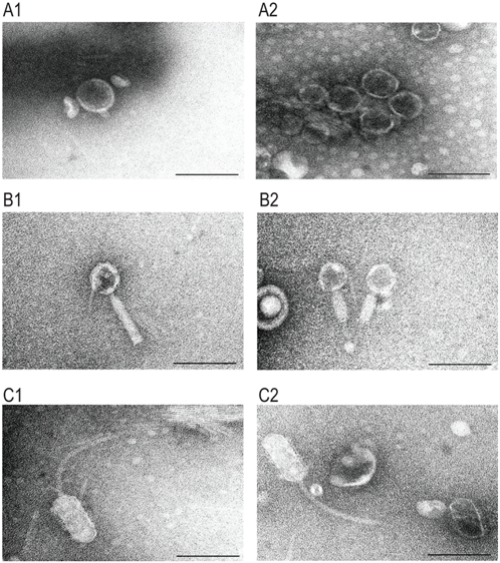
Electron micrographs of phage particles produced by O157 Sakai. Electron micrographs of three types of phage particles detected in the culture supernatant of O157 Sakai are shown. (A) Phage particles with a short tail. (B) Phage particles with a contractile and non-flexible tail. (C) Phage particles with an elongated head and a long flexible tail. Phage particles shown in (A) are derived from Sp5 because K-12 strains lysogenized by CmR-marked Sp5 produced phage particles with the identical morphology. The similarity to phage Mu suggests that the phage particles shown in (B) are derived from Sp18. Electron micrographs were taken at magnifications of 60 K or 80 K using a transmission electron microscope and negatively contrasted with 2% uranyl acetate dihydrate. Bar, 100 nm.

Finally, we performed an electron microscopic examination of phage particles that were present in the culture supernatants of MMC-treated and untreated O157 Sakai cells (Figure 7). The MMC-treated sample contained numerous phage particles with a short tail attached to a head approximately 56 nm in diameter (Figure 7A). The dominant induction of Sp5 by MMC treatment (Figure 4) suggests that these phage particles originated from Sp5. In fact, K-12 strains lysogenized by Cm^R^-marked Sp5 produced phage particles with the identical morphology. The morphology of Sp5 (Figure 7A) is highly similar to that of the previously reported Stx2 phage of O157 EDL933 [27]. We were unable to detect other phage types in the MMC-treated sample. However, in addition to Sp5, at least two other types of phage particles were detected in the untreated sample. The second type had a head with a hexagonal outline approximately 49 nm in diameter, which was connected by a neck to a contractile and non-flexible tail (the uncontracted sheath is approximately 100 nm long and the contracted one is 55 nm) (Figure 7B). The similarity to the morphology of Mu phage particles indicates that this second type most likely originated from Sp18. The third type had a head with an elongated hexagonal outline (44 wide and 95 nm long) and a 147 nm long flexible tail (Figure 7C). This phage probably derived from some of the lambdoid prophages, but its origin is difficult to pinpoint because lambdoid prophages other than Sp5 (including Sp10 and Sp6) contain very similar morphogenetic genes (Figure 1).

We also examined the culture supernatants of K-12 strains lysogenized with Cm^R^ derivatives of Sp6 and Sp10 for the production of phage particles, but no phage particle was detected in the culture supernatants of Sp6 and Sp10 lysogens (data not shown).

## Discussion

The results of our *in silico* analysis of the potential activities of 18 prophages on the O157 Sakai genome indicate that all but Sp5 contain one or more genetic defects (Figure 1, Figure S1, and Table 1). This suggests that the present-day O157 prophage pool may have low potential activity as mobile genetic elements to spread virulence genes, although their mobility in evolutionary history has played an essential role in the emergence of this highly virulent *E. coli* lineage. Nevertheless, our systematic experimental evaluation of the Sps revealed that many have unexpectedly high potential activity to function as mobile genetic elements. First, nine Sps could excise themselves from the chromosome and replicate in the O157 cells in response to spontaneous or MMC-mediated induction (Figure 3, Figure 4, and Table 2). They can be divided into three groups according to their induction patterns: (i) spontaneously inducible (Sp6, Sp7, Sp9, Sp10, and Sp18), (ii) spontaneously inducible and further enhanced by MMC-mediated induction (Sp5, Sp13, and Sp15), and (iii) inducible only by the regional replication of other prophages (Sp4 by Sp5 and Sp14 by Sp15). Second, most of these Sps, except Sp6 and Sp14, were packaged into phage particles (Figure 4), although half of them (Sp4, Sp9, Sp13, and Sp15) contain defects in head formation or DNA packaging. Third, we found that the Cm^R^ gene cassette on four Sps is transferable to other *E. coli* strains, although we used only two K-12 derivatives as recipients (Table 2). Three (Sp5, Sp6, and Sp10) were transferred to K-12 and stably lysogenized in the chromosome (Figure 5). This result indicates that Sp6, which is also defective in head and tail formation, can be packaged, although this was not clear from the particulate DNA quantification by qPCR (Figure 4). It is also important that these three phages carry several important virulence determinants, including the *stx2* genes and multiple non-LEE effector genes (Figure 1 and Figure S1). Fourth, the Cm^R^ gene cassette inserted into the Sp15 genome by replacing the *stx1* gene was transferred to K-12, and this transfer was achieved through the generation of a chimera between Sp15 and Sp5 (Figure 6 and Figure S7). In addition, three types of phage with distinct morphologies were detected in the culture supernatant of O157 Sakai (Figure 7). Two derive from Sp5 and Sp18, respectively, but the origin of the third remains undetermined. These results indicate that many apparently defective prophages of O157 Sakai should not be regarded as simple phage remnants, but rather as active genetic elements that can potentially mediate or assist HGT of various virulence determinants encoded on the O157 genome. The results further suggest that various types of inter-prophage interactions occur in the O157 prophage pool, and these interactions induce the biological activities of the defective prophages.

Inter-prophage interactions that most likely occurred in the O157 prophage pool complemented various defects in morphogenetic functions by providing the proteins for phage particle formation. Although the details of this complementation remain to be elucidated, the lambdoid phages on the O157 genome share nearly identical morphogenetic genes [11],[12] in various combinations (Figure 1). They therefore appear capable of supplying virion proteins compatible with those of other lambdoid phages. In fact, we identified one type of phage particle that differs from that of Sp5 but has lambdoid features in the culture supernatant of O157 Sakai (Figure 7C). In some cases, whole virion proteins may be provided by other prophages; this may be the situation with Sp7 and Sp13. Both have severe defects in morphogenetic functions, but are nevertheless packaged. Furthermore, because Sp13 is the only member of the P2-like phage family in the O157 prophage pool, this type of inter-prophage interaction may occur between very different types of bacteriophages.

In the case of Sp7, another type of interaction may complement its defect in replication function. This highly degraded prophage lacks most morphogenesis genes, as well as repressor and antirepressor genes. Furthermore, the replication gene, which resembles the P4 *α* gene, has been disrupted into three fragments (Figure 1E and Figure S1). Nevertheless, Sp7 is spontaneously inducible and a significant amount of circularized DNA was observed to accumulate in the O157 cells (Figure 4). Thus, the replication of Sp7 may be mediated by the replication proteins of Sp13 (P2-like phage) or Sp2 (P4-like phage), although we cannot exclude the possibility that some (or all) of the fragmented polypeptides of Sp7 may still contain some replication initiation activity.

Replication or amplification of the Sp4 and Sp14 genomes is another type of inter-prophage interaction. Their genomic DNA can be amplified only by the regional replication of Sp5 and Sp15, respectively. Although both lack the genes for excisionase, integrases alone appear capable of mediating their excision from the chromosome. Thus, the two prophage genomes amplified by regional replication are excised into a circularized form (Figure 3 and Figure 4). More interestingly, although Sp4 does not encode an intact packaging enzyme (terminase) by itself (Figure 1 and Figure S1), its amplified genome was found to be packaged (Figure 4). Most likely, this packaging was carried out by the terminase of Sp14, because the putative *cos* sequence of Sp4, which needs to be digested by terminase for packaging, is nearly identical to that of Sp14 (Figure S8).

Finally, the recombination between Sp15 and Sp5 can also be regarded as an inter-prophage interaction because it occurred in the O157 prophage pool and generated new Stx1-tranducing phages (Figure 5 and Figure S7). This type of inter-prophage interaction can occur between other lambdoid prophages of O157 as well, because they share nearly identical sequences, which can therefore recombine [11],[12]. Similar recombination may also occur between the resident prophages and newly incoming phages. In this way, high levels of excision and replication of defective prophage genomes in O157 cells may provide significant opportunities for such recombination. This may explain why a surprisingly high level of structural variation is observed in the prophage regions among O157 genomes [14],[45], which, in turn, supports the hypothesis that O157 cells function as “phage factories” that produce a wide variety of bacteriophages in nature [12].

In conclusion, many of the prophages of O157 Sakai that contain a wide range of genetic defects show unexpectedly high potential activity as mobile genetic elements, and this mobility is probably achieved through various types of inter-prophage interactions that occur in the O157 prophage pool. Thus, these apparently defective prophages are not simply remnants generated in the course of O157 evolution, but instead should be regarded as genetic elements that are potentially capable of spreading virulence determinants and other genetic traits to other bacterial strains. Similarly to *E. coli*, many other bacteria contain multiple prophages with genetic defects, and the potential of these sequence elements to function as mobile elements has been largely ignored. Our findings suggest that more attention should be paid to their potential roles in HGT between bacteria and in the evolution of bacterial pathogens.

## Materials and Methods

### Bacterial strains, growth conditions, and extraction of total cellular and phage DNA

O157 Sakai (RIMD 050995) was isolated in a large outbreak that occurred in Sakai city, Japan, in 1996 [46], and the complete genome sequence has been determined [11]. Cells were grown overnight to stationary phase at 37°C in Luria-Bertani (LB) medium. For prophage induction with MMC, cells were grown to early log phase (OD_600_ 0.2–0.4), and MMC was added to the culture to a final concentration of 1 µg/ml. At 1-hr intervals, we isolated aliquots of the culture and collected the cells by centrifugation at 4°C. Total cellular DNA was isolated from the cells using the Genomic-tip 100/G and the Genomic DNA buffer set (Qiagen, CA, USA) according to the manufacturer's instructions.

Phage particles were isolated from the culture supernatants 10 hr after the addition of MMC. The culture was first treated with chloroform, and bacterial cell debris was removed by centrifugation. The supernatant was filtered through 0.22-µm pore-size filters (Millipore Corp., MA, USA) and incubated with 200 U/ml DNase I (Invitrogen, CA, USA) at 37°C for 1 hour. After incubation, 0.25 volumes of a solution containing 20% polyethylene glycol 8000 (PEG) and 10% NaCl was added to the sample. The mixture was kept at 4°C overnight and then centrifuged at 12,000×*g* for 1 hour to precipitate phage particles. The phage particles were suspended in SM buffer (0.58% NaCl, 0.2% MgSO_4_⋅7H_2_O, 1 M Tris-Cl (pH 7.5), 0.01% gelatin) and incubated with DNase I (final concentration, 1000 U/ml) and RNase A (50 µg/ml) (Stratagene, CA, USA) at 37°C for 1 hour. After DNase and RNase treatment, the sample was treated with proteinase K (100 µg/ml; Wako, Osaka, Japan) at 50°C for 1 hour, and phage DNA was isolated using the Genomic-tip 20/G. Total cellar DNA and phage DNA from untreated samples were prepared by the same protocol, except that no MMC was added to the culture.

### Comparative bioinformatic analysis of prophages

Comparative analysis of O157 Sakai prophage genomes was performed using the “in silico MolecularCloning(R) (IMC)” software (Genomics edition, version 1.4.71, In Silico Biology, Inc., Kanagawa, Japan). Homology searches were performed using BLAST2 [47], and functional motifs were searched using InterProScan (http://www.ebi.ac.uk/Tools/InterProScan/). Multiple protein sequence alignment was carried out using CLUSTALW [48] and analyzed by the multiple alignment editor Jalview (http://www.ebi.ac.uk/clustalw/). A neighbor-joining tree for replication elongation proteins was generated using MEGA3 [49].

### Total RNA isolation

An overnight culture in LB medium was diluted to an OD_600_ of 0.2 and grown for 1 hour. At this point (0 min), MMC was added to the culture at a concentration of 1 µg/ml. Samples were collected at 0-min, 45-min and 90-min intervals from the cultures treated with MMC or those left untreated. The RNAprotect Bacteria Reagent (QIAGEN, Valencia, CA) was immediately added to the samples, and total RNA was isolated using the RNeasy Plus Mini kit (QIAGEN, Valencia, CA) according to the manufacturer's instructions. RNA quality was assessed by spectrophotometry using the NanoDrop instrument (NanoDrop Technologies, Inc., USA) and by agarose gel electrophoresis.

### Microarray design

Probes (60 mer) were designed for the 5,447 protein-coding genes of the O157 Sakai genome. The O157 Sakai genome contains many multi-copy genes that are derived from IS elements and lambdoid prophages sharing nearly identical sequences. Thus, to avoid effects due to cross hybridization, all data for the probes that showed >80% DNA sequence identity to any other genomic regions of the O157 Sakai genome were removed from the data set. Finally, we used 4,507 probes representing 4,507 genes. Among the 4,507 probes, 452 were for the genes on 18 prophage regions. Arrays were produced by Agilent Technologies (Palo Alto, CA, USA) by the *in situ* oligonucleotide synthesis method.

### Labeling, hybridization, scanning, and data analysis

For DNA microarray analyses, test and reference DNA (250 ng) were chemically labeled with ULS-Cy3 and ULS-Cy5, respectively, using the Agilent Oligo CGH Microarray Kit (Agilent Technologies). The fluorescently labeled DNA was purified by the Agilent KREApure column. The Cy5-labeled and Cy3-labeled DNA were mixed and used for hybridization. For RNA analysis, total RNA (10 µg) was reverse transcribed and labeled with amino-allyl dUTP using MMLV-RT (Agilent Technologies) and random hexamers (Invitrogen). The cDNA from test and control samples was labeled with Cy3 and Cy5 dye, respectively (Monofunctional NHS-ester Dye, Amersham). The Cy3-labeled and Cy5-labeled cDNAs were purified, combined, and used for hybridization. The arrays were scanned using an Agilent scanner (Agilent Technologies), and data extraction, filtering and normalization were conducted using Feature Extraction software (Agilent Technologies) according to the manufacturer's instructions. Each sample was examined twice using the labeled DNAs and cDNAs independently prepared for each hybridization. Data analysis and visualization were done using Microsoft Excel and MultiExperiment Viewer (The Institute for Genome Research) [50].

### PCR and DNA Sequencing of PCR products

PCR was carried out using an Ex-taq PCR amplification kit (Takara Bio, Kyoto, Japan). The PCR cycling program consisted of 29 cycles of 45 sec at 95°C, 45 sec at 60°C, and 1 min at 72°C, with an additional step of 2 min at 72°C. Sequencing of the PCR products was carried out using an ABI PRISM 3100 automated sequencer (PE Biosystems, CA, USA). Primers designed for PCR amplification were used as sequencing primers. Sequencher™ software (version 4.2.2, Gene Codes Corporation, MI, USA) was used for sequence data analyses. All primers used are listed in Table S1.

### Quantitative PCR

TaqMan probes and PCR primers for real-time qPCR (Table S1) were designed using Primer Express software (Primer Express™, PE BioSystems) according to the manufacturer's instructions. All analyses were performed using the ABI PRISM 7000 Sequence Detection System (PE BioSystems). To analyze the intracellular prophage DNA, 10 ng of total cellular DNA was used as template DNA in a 50 µl reaction volume. To analyze the phage particle DNA, phage DNA isolated from 1 ml (MMC-treated samples) or 50 ml (untreated samples) of culture supernatants was used as the template. Primers and TaqMan probes were used at a concentration of 400 nM and 250 nM, respectively. FAM (5′) and TAMRA (3′) were used as reporter and quencher dyes for TaqMan probes, respectively. The PCR cycling program consisted of 45 cycles of 15 sec at 95°C and 1 min at 60°C. For quantification of DNA, the standard curve method was employed. Standard curves were constructed over the range from 10^3^ copies/µl to 10^7^ copies/µl for each amplicon. Quantities of two chromosomal backbone regions (CB1 and CB2 in Figure 3 and Figure 4) were monitored as controls in qPCR analyses. The concentrations of chromosomal backbone DNA measured in the total cellular DNA preparations were comparable with those estimated from the numbers of cells used for DNA preparation (Figure 3 and Figure 4), indicating the validity of the real-time qPCR assays.

### FIGE analysis of phage DNA

FIGE analysis of packaged phage DNA was performed using phage particles collected from culture supernatants by PEG/NaCl precipitation. Precipitated phage particles were embedded in plugs of 1% Certified Low Melt Agarose (Bio-Rad Laboratories, Inc., CA, USA) and treated at 37°C for 2 hr with DNase I (1000 U/ml) and RNase A (50 µg/ml) in DNase I buffer (10 mM Tris (pH 7.5), 2.5 mM MgCl_2_, and 0.5 mM CaCl_2_), followed by overnight incubation with proteinase K (100 µg/ml) at 50°C in TE containing 1% sodium dodecyl sulfate. After the plugs were washed three times with TE buffer at 15-minute intervals, the plugs were sliced into appropriate sizes and subjected to FIGE analysis. FIGE was performed using a CHEF MAPPER (Bio-Rad Laboratories) with a 1% agarose gel, and initial and final switch times of 0.11 and 0.92 sec, respectively. Total run time was 20.3 h with 9.0 V/cm (forward) and 6.0 V/cm (reverse) voltage at a constant temperature of 14°C. The gel was stained with ethidium bromide to visualize DNA bands.

### Analysis of transfer and lysogenization of prophages into K-12 strains

The Cm^R^ cassette was inserted into eight Sps (Figure 5A) by replacing moron genes on each prophage genome using the method described by Datsenko and Wanner [51]. Primer sequences utilized for gene replacement and confirmation of replacement are listed in Table S2. In Sp5 (Stx2 phage) and Sp15 (Stx1 phage), the entire *stx* genes were replaced by the Cm^R^ cassette. In other Sps, genes for T3SS effectors or other morons were selected as targets for gene replacement. Therefore, the O157 Sakai derivatives generated in this study are predicted to have reduced potential virulence.

Cm^R^-O157 Sakai derivatives (Table S3) were grown at 37°C overnight in LB containing 40 µg/ml of Cm. The cells were subcultured to an OD_600_ of 0.2–0.4 in LB without antibiotic and then cultivated at 37°C for 10 hrs with vigorous shaking in the presence or absence of MMC (10 µg/ml). Phage particles in the culture supernatants were recovered by PEG/NaCl precipitation and suspended in SM buffer. The precipitated phage preparations were gently treated with 0.1 volumes of chloroform. After brief centrifugation, aqueous phases of each sample were collected and incubated at 37°C for 15 min to remove residual chloroform. Phage solution prepared from a 20-ml culture supernatant of each O157 Sakai derivative was incubated with 10^8^ recipient cells suspended in 100 µl of the SM buffer at 28°C for 1 hour. Finally, the recipient cells were plated on LB agar plates containing Cm (50 µg/ml) and incubated overnight at 37°C. Randomly selected colonies were checked by the agglutination test using anti-O157 serum to ensure that all were derived from K-12 strains; colonies were subsequently used in further analyses. The Cm^R^ transductants obtained were verified for the lysogeny of Cm^R^-marked prophages using PCR (Figure 5). Primers used are listed in Table S4.

To analyze the chimeric phages of Sp15Δ*stx1*::Cm^R^ and Sp5, PCR scanning analysis of the *stx1*-flanking region was performed using eight primer pairs (Figure 6A, Figure S7, and Table S5). To confirm the chimeric structure of the recombinant phage, we performed two types of PCR analyses, one using the forward (P_F) and reverse (4R) primers specific to the *P* gene of Sp5 and the Cm^R^ gene cassette, respectively, and another using the forward (6F) and reverse (T_R) primers specific to the Cm^R^ gene cassette and the Sp5 *nu1* gene, respectively. These primer sequences are also listed in Table S6. Integration of an Sp5-like phage into the *wrbA* locus in Sp15Δ*stx1*::Cm^R^-transductants was confirmed by PCR using two primer pairs (Table S4) to amplify the left (primers LbF and LbR) and right (RbF and RbR) attachment sites of Sp5 (Figure 6C and Figure S7).

### Electron microscopy

Phage particles were collected by PEG/NaCl precipitation from the culture supernatants of MMC-treated or untreated O157 Sakai and suspended in SM buffer. A 10-µl drop of the suspension was placed on copper grids with carbon-coated Formvar films and negatively contrasted with 2% uranyl acetate dihydrate. Samples were examined using a transmission electron microscope (1200EX, JEOL, Tokyo) operated at 80 kV. Average dimensions were measured with the image processing and analysis software ImageJ (http://rsb.info.nih.gov/ij/).

### ID numbers for genes mentioned in the text

All sequence information for prophages and genes of the O157 Sakai genome is available at the DDBJ/EMBL/NCBI database (accession no. BA000007)

## Supporting Information

Figure S1 Genetic structures of 18 Sakai prophages and their alignment with the corresponding prototype phage genomes (enlarged version of Figure 1). An enlarged version of Figure 1 with more detailed information is shown.
(0.87 MB PDF)

Figure S2 Schematic presentation of the domain structures of repressors and antirepressors of O157 Sps and their corresponding prototype phages. Functional motifs identified in the predicted repressors and antirepressors of Sps are shown. Motifs in repressors and antirepressors of prototype phages and their relatives are also shown.
(0.07 MB PDF)

Figure S3 Replication elongation proteins of O157 Sakai lambdoid prophages. Amino acid sequences of replication elongation proteins of lambdoid Sps were compared with those of reference proteins from other lambda phage family members. A neighbor-joining (N-J) tree constructed based on the sequence comparisons is shown. Reference sequences are from phage lambda (λ), enterobacteria phage P22 (P22), phage BP4795 (BP), bacteriophage phi80 (phi80), K-12 cryptic prophage Rac (Rac), enterobacteria phage HK022 (HK022), *Salmonella* phage Gifsy-2 (G2), and enterobacteria phage ST64T (ST64T), *E. coli* DnaB (DnaB_Ec), and *E. coli* DnaC (DnaC_Ec).
(0.07 MB PDF)

Figure S4 Cell lysis of MMC-treated O157 Sakai cells. Growth of O157 Sakai cells in the absence (−MMC) or presence (+MMC) of MMC was monitored by measuring the OD_600_ of each culture. Time points of sample collection for microarray analysis (Figure 2) are indicated. MMC was added to the culture at the 0-h point.
(0.04 MB PDF)

Figure S5 Attachment site (*attP*) sequences of Sakai prophages. DNA sequences of attachment sites (*attP*) of the nine Sps that were found to be excised and circularized in O157 Sakai cells are shown. Right (R) and left (L) junctions of prophage regions on the O157 Sakai chromosome are aligned with the attP sequence determined in this study (P). Core attachment site sequences are indicated in blue. Nucleotide positions on the O157 Sakai chromosome are also indicated for right and left junctions.
(0.10 MB PDF)

Figure S6 Ratios of MMC-induced and spontaneously induced prophage genomes. Ratios of circularized prophage genomes in MMC-treated O157 Sakai cells to those in untreated cells are shown for each of the nine Sps that were found to be excised and circularized in O157 Sakai cells. As controls, amounts of the Sp18 DNA and chromosomal DNA from two regions (chromosomal backbone, CB1 and CB2) were measured. Note that Sp18 is not inducible by MMC treatment, and its genome is not circularized, similar to results previously reported for the prototype Mu phage.
(0.04 MB PDF)

Figure S7 Sequence homology between Sp5 and Sp15 genomes and PCR scanning analysis of Sp5/Sp15 recombinant phages. Gene organizations of the *stx1* - and *stx2*-flanking regions of Sp15 and Sp5 are shown. The *stx1* gene of Sp15 has been replaced by the Cm^R^ cassette. Highly homologous regions (>90% nucleotide sequence identity) that probably mediated the recombination between the Sp5 and Sp15 genomes are indicated by gray shading. PCR primer positions used for PCR scanning analysis of Sp5/Sp15 recombinant phages (RP) are indicated by arrows. The results of PCR amplification shown in Figure 6 of the main text are indicated by solid (amplified) or dotted (not amplified) lines between the primer pair.
(0.13 MB PDF)

Figure S8 Sequence alignments of the *cos* sequences and the Nu1 subunit proteins of Sp4, Sp14, and phage lambda. (A) DNA sequences of the cos sites of Sp4 and Sp14 are identical. (B) Amino acid sequences of the Nu1 proteins of Sp4 and Sp14 are also identical, but the corresponding sequence in Sp4 has been disrupted by an IS*Ec8*-insertion.
(0.10 MB PDF)

Table S1 Primers and TaqMan probes for qualitative PCR and real-time qPCR.
(0.08 MB DOC)

Table S2 Primers used to construct Cm^R^-marked prophages and verify the insertion of the Cm^R^ gene cassette.
(0.16 MB DOC)

Table S3 Bacterial strains and plasmids used in this study.
(0.06 MB DOC)

Table S4 Primers used to examine integration and lysogenization of Sakai prophages in K-12 strains.
(0.08 MB DOC)

Table S5 Primers used to analyze the Sp15/Sp5 recombinant phage.
(0.05 MB DOC)

## References

[1] Brüssow H, Canchaya C, Hardt W D (2004). Phages and the Evolution of Bacterial Pathogens: from Genomic Rearrangements to Lysogenic Conversion.. Microbiol Mol Biol Rev.

[2] Filee J, Forterre P, Laurent J (2003). The role played by viruses in the evolution of their hosts: A view based on informational protein phylogenies.. Res Microbiol.

[3] Canchaya C, Proux C, Fournous G, Bruttin A, Brüssow H (2003). Prophage Genomics.. Microbiol Mol Biol Rev.

[4] Freeman VJ (1951). Studies on the virulence of bacteriophage-infected strains of Corynebacterium diphtheriae.. J Bacteriol.

[5] Casjens S (2003). Prophages and bacterial genomics: What have we learned so far?. Mol Microbiol.

[6] Hacker J, Carniel E (2001). Ecological fitness, genomic islands and bacterial pathogenicity. A Darwinian view of the evolution of microbes.. EMBO Rep.

[7] Wagner PL, Waldor MK (2002). Bacteriophage control of bacterial virulence.. Infect Immun.

[8] Canchaya C, Fournous G, Brüssow H (2004). The impact of prophages on bacterial chromosomes.. Mol Microbiol.

[9] Paton JC, Paton AW (1998). Pathogenesis and diagnosis of Shiga toxin-producing *Escherichia coli* infections.. Clin Microbiol Rev.

[10] Hayashi T, Ooka T, Ogura Y, Asadulghani, Baquero F, Nombela C, Cassell GH, Gutierrrez JA (2007). Genomic View on the Evolution of Enterohemorrhagic *Escherichia coli*.. Evolutionary Biology of Bacterial and Fungal Pathogens.

[11] Hayashi T, Makino K, Ohnishi M, Kurokawa K, Ishii K (2001). Complete genome sequence of enterohemorrhagic *Escherichia coli* O157: H7 and genomic comparison with a laboratory strain K-12.. DNA Res.

[12] Ohnishi M, Kurokawa K, Hayashi T (2001). Diversification of *Escherichia coli* genomes: are bacteriophages the major contributors?. Trends Microbiol.

[13] Tobe T, Beatson SA, Taniguchi H, Abe H, Bailey CM (2006). An extensive repertoire of type III secretion effectors in *Escherichia coli* O157 and the role of lambdoid phages in their dissemination.. Proc Natl Acad Sci U S A.

[14] Ogura Y, Kurokawa K, Ooka T, Tashiro K, Tobe T (2006). Complexity of the Genomic Diversity in Enterohemorrhagic *Escherichia coli* O157 Revealed by the Combinational Use of the O157 Sakai OligoDNA Microarray and the Whole Genome PCR scanning.. DNA Res.

[15] Muniesa M, Blanco JE, De Simon M, Serra-Moreno R, Blanch AR (2004). Diversity of stx2 converting bacteriophages induced from Shiga-toxin-producing *Escherichia coli* strains isolated from cattle.. Microbiology.

[16] Svenningsen SL, Costantino N, Court DL, Adhya S (2005). On the role of Cro in lambda prophage induction.. Proc Natl Acad Sci U S A.

[17] Little JW (2005). Threshold effects in gene regulation: when some is not enough.. Proc Natl Acad Sci U S A.

[18] Hendrix RW, Casjens S, Calendar R (2005). Bacteriophage λ and its Genetic Neighborhood.. The Bacteriophages.

[19] Kobiler O, Rokney A, Friedman N, Court DL, Stavans J (2005). Quantitative kinetic analysis of the bacteriophage lambda genetic network.. Proc Natl Acad Sci U S A.

[20] Van Duyne GD (2005). Lambda integrase: armed for recombination.. Curr Biol.

[21] Sam MD, Papagiannis CV, Connolly KM, Corselli L, Iwahara J (2002). Regulation of directionality in bacteriophage lambda site-specific recombination: structure of the Xis protein.. J Mol Biol.

[22] Skalka AM (1977). DNA Replication—Bacteriophage lambda.. Curr Top Microbiol Immunol.

[23] Konieczny I (2003). Strategies for helicase recruitment and loading in bacteria.. EMBO Rep.

[24] Mallory JB, Alfano C, McMacken R (1990). Host virus interactions in the initiation of bacteriophage lambda DNA replication. Recruitment of *Escherichia coli* DnaB helicase by lambda P replication protein.. J Biol Chem.

[25] Catalano CE (2000). The terminase enzyme from bacteriophage lambda: a DNA-packaging machine.. Cell Mol Life Sci.

[26] Gaussier H, Yang Q, Catalano CE (2006). Building a virus from scratch: assembly of an infectious virus using purified components in a rigorously defined biochemical assay system.. J Mol Biol.

[27] Schmidt H (2001). Shiga-toxin-converting bacteriophages.. Res Microbiol.

[28] Iguchi A, Iyoda S, Watanabe H, Osawa R (2006). O Side Chain Deficiency Enhances Sensitivity of *Escherichia coli* to Shiga Toxin 2-Converting Bacteriophages.. Current Microbiol.

[29] Lindqvist BH, Deho G, Calendar R (1993). Mechanisms of genome propagation and helper exploitation by satellite phage P4.. Microbiol Rev.

[30] Tao Liu, Renberg SaraK, Haggard-Ljungquist E (1998). The E protein of satellite phage P4 acts as an antirepressor by binding to the C protein of helper phage P2.. Mol Microbiol.

[31] Eriksson SK, Liu T, Haggard-Ljungquist E (2000). Interacting interfaces of the P4 antirepressor E and the P2 immunity repressor C.. Mol Microbiol.

[32] Wang S, Chang JR, Dokland T (2006). Assembly of bacteriophage P2 and P4 procapsids with internal scaffolding protein.. Virology.

[33] Kim KJ, Song J (2006). Isolation and characterization of the smallest bacteriophage P4 derivatives packaged into P4-size head in bacteriophage P2-P4 system.. J Microbiol.

[34] Howe MM, Bade EG (1975). Molecular biology of bacteriophage Mu.. Science.

[35] Paolozzi L, Ghelardini P, Calendar R (2005). Bacteriophage Mu.. The Bacteriophages.

[36] Cali S, Spoldi E, Piazzolla D, Dodd IB, Forti F (2004). Bacteriophage P4 Vis protein is needed for prophage excision.. Virology.

[37] Fukasawa T, Hirai K, Segawa T, Obonai K (1978). Regional replication of the bacterial chromosome induced by derepression of prophage lambda. IV. Escape synthesis of *gal* operon in phage 82.. Mol Gen Genet.

[38] Frye JG, Porwollik S, Blackmer F, Cheng P, McClelland M (2005). Host gene expression changes and DNA amplification during temperate phage induction.. J Bacteriol.

[39] Courcelle J, Khodursky A, Peter B, Brown PO, Hanawalt PC (2001). Comparative gene expression profiles following UV exposure in wild-type and SOS-deficient Escherichia coli.. Genetics.

[40] Khil PP, Camerini-Otero RD (2002). Over 1000 genes are involved in the DNA damage response of *Escherichia coli*.. Mol Microbiol.

[41] Lindroos H, Vinnere O, Mira A, Repsilber D, Naslund K (2006). Genome Rearrangements, Deletions and Amplifications in the Natural Population of *Bartonella henselae*.. J Bacteriol.

[42] McAdams HH, Shapiro L (1995). Circuit simulation of genetic networks.. Science.

[43] Kelley WL (2006). Lex marks the spot: the virulent side of SOS and a closer look at the LexA regulon.. Mol Microbiol.

[44] Waldor MK, Friedman DI (2005). Phage regulatory circuits and virulence gene expression.. Curr Opin Microbiol.

[45] Ohnishi M, Terajima J, Kurokawa K, Nakayama K, Murata T (2002). Genomic diversity of enterohemorrhagic *Escherichia coli* O157 revealed by whole genome PCR scanning.. Proc Natl Acad Sci U S A.

[46] Watanabe H, Wada A, Inagaki Y, Itoh K, Tamura K (1996). Outbreaks of enterohaemorrhagic *Escherichia coli* O157:H7 infection by two different genotype strains in Japan, 1996.. Lancet.

[47] Wang H, Ooi BC, Tan KL, Ong TH, Zhou L (2003). BLAST++: BLASTing queries in batches.. Bioinformatics.

[48] Thompson JD, Higgins DG, Gibson TJ (1994). CLUSTAL W: improving the sensitivity of progressive multiple sequence alignment through sequence weighting, position-specific gap penalties and weight matrix choice.. Nucleic Acids Res.

[49] Kumar S, Tamura K, Nei M (2004). MEGA3: integrated software for molecular evolutionary genetics analysis and sequence alignment.. Brief Bioinform.

[50] Saeed AI, Sharov V, White J, Li J, Liang W, Bhagabati N (2003). TM4: a free, open-source system for microarray data management and analysis.. Biotechniques.

[51] Datsenko KA, Wanner BL (2000). One-step inactivation of chromosomal genes in *Escherichia coli* K-12 using PCR products.. Proc Natl Acad Sci U S A.

