# Supplementary figures and images for: The Defective Prophage Pool of Escherichia coli O157: Prophage–Prophage Interactions Potentiate Horizontal Transfer of Virulence Determinants
Source: PLoS Pathog. 2009 May 1;5(5):e1000408. doi: 10.1371/journal.ppat.1000408 (PMC2669165; doi:10.1371/journal.ppat.1000408)

A

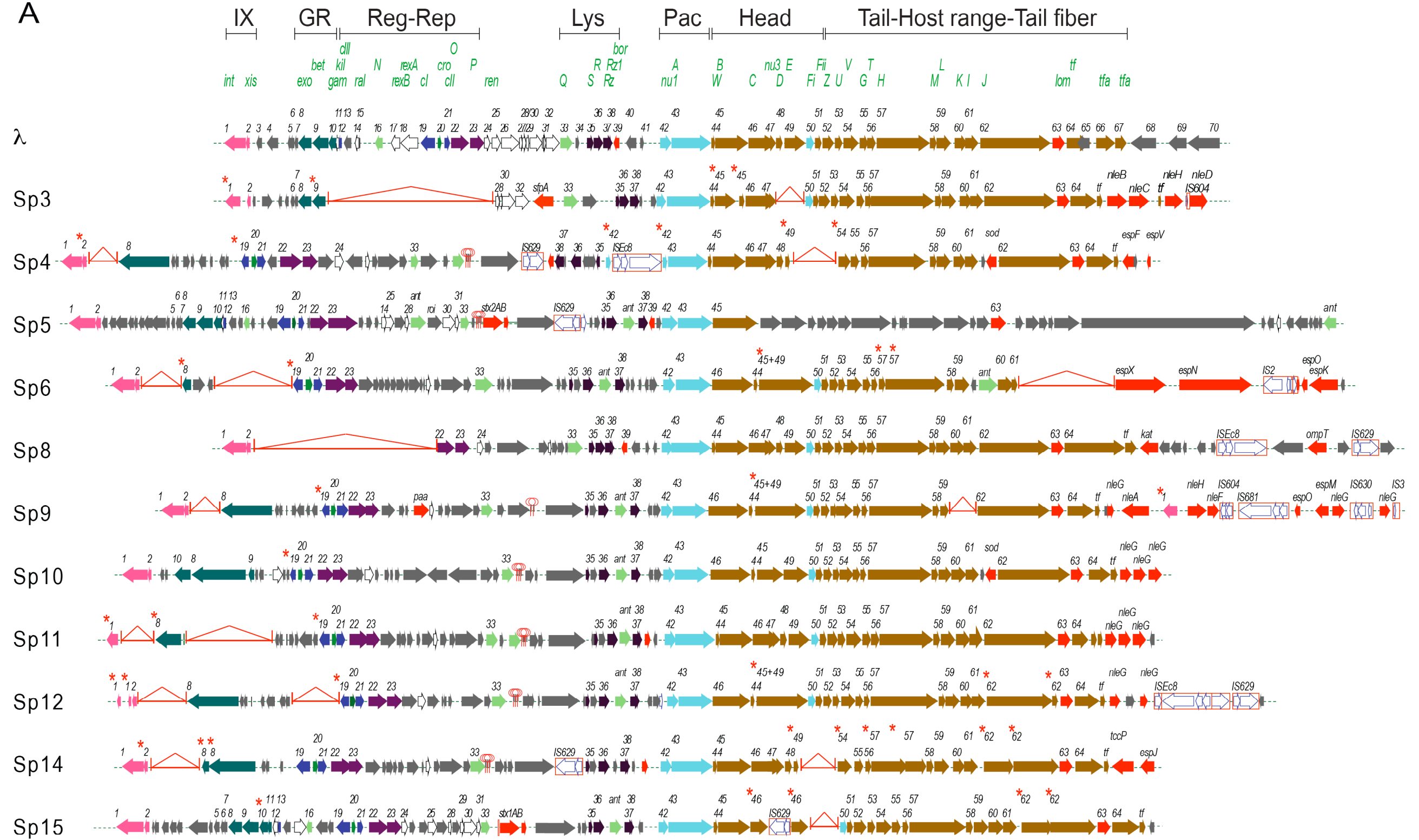

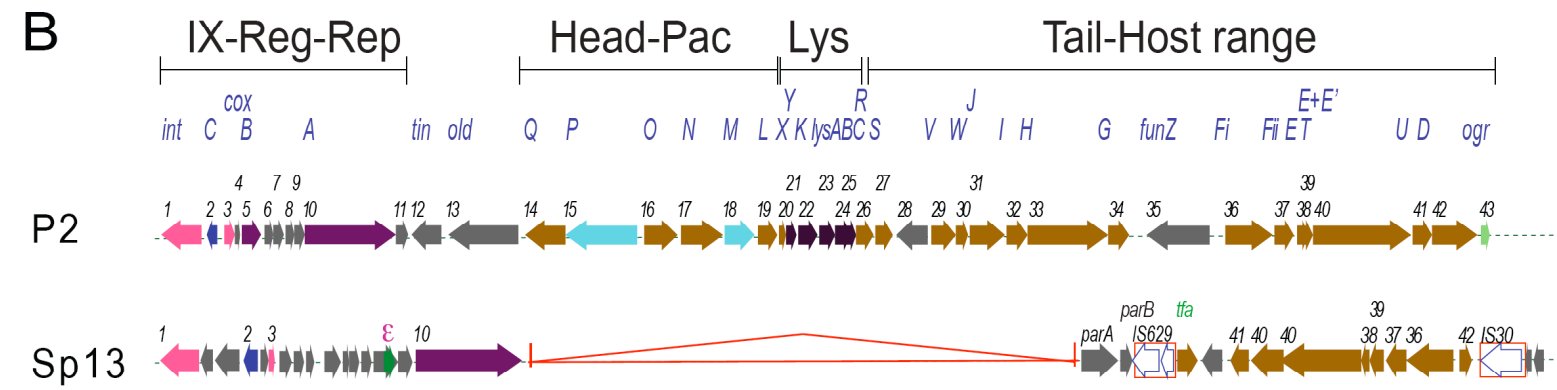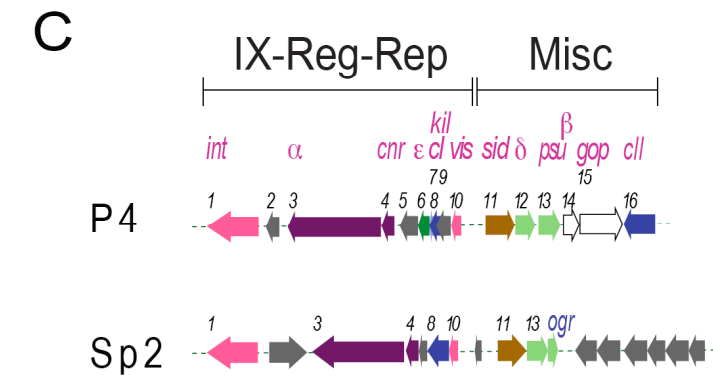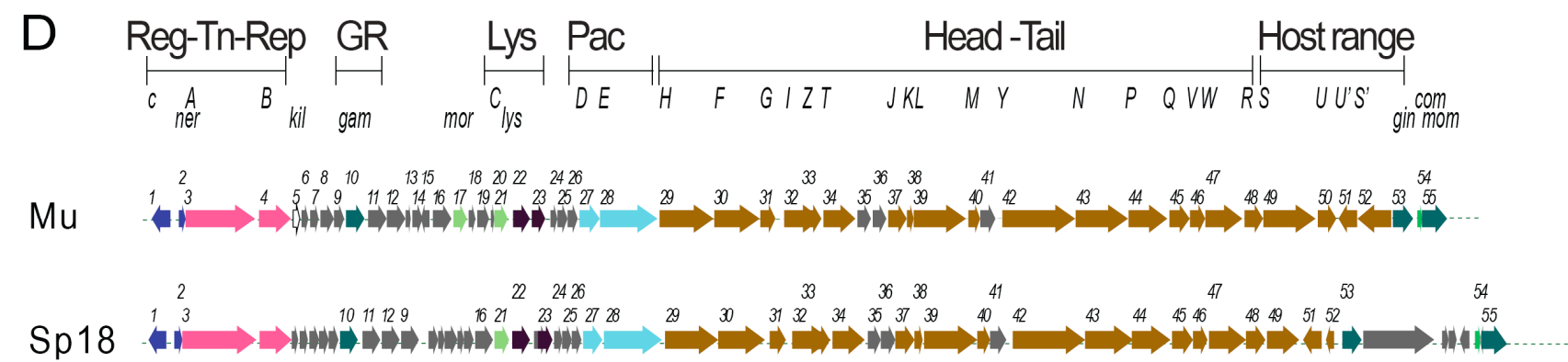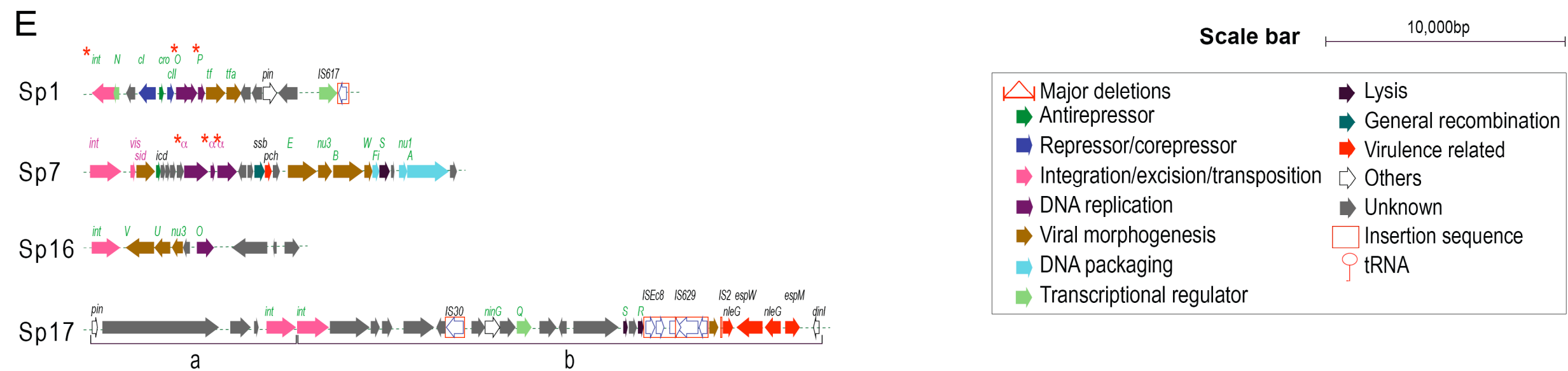

Supplement: Figure S1 — Genetic structures of 18 Sakai prophages and their alignment with the corresponding prototype phage genomes (enlarged version of Figure 1). An enlarged version of Figure 1 with more detailed information is shown. (0.87 MB PDF) [file ppat.1000408.s001.pdf]

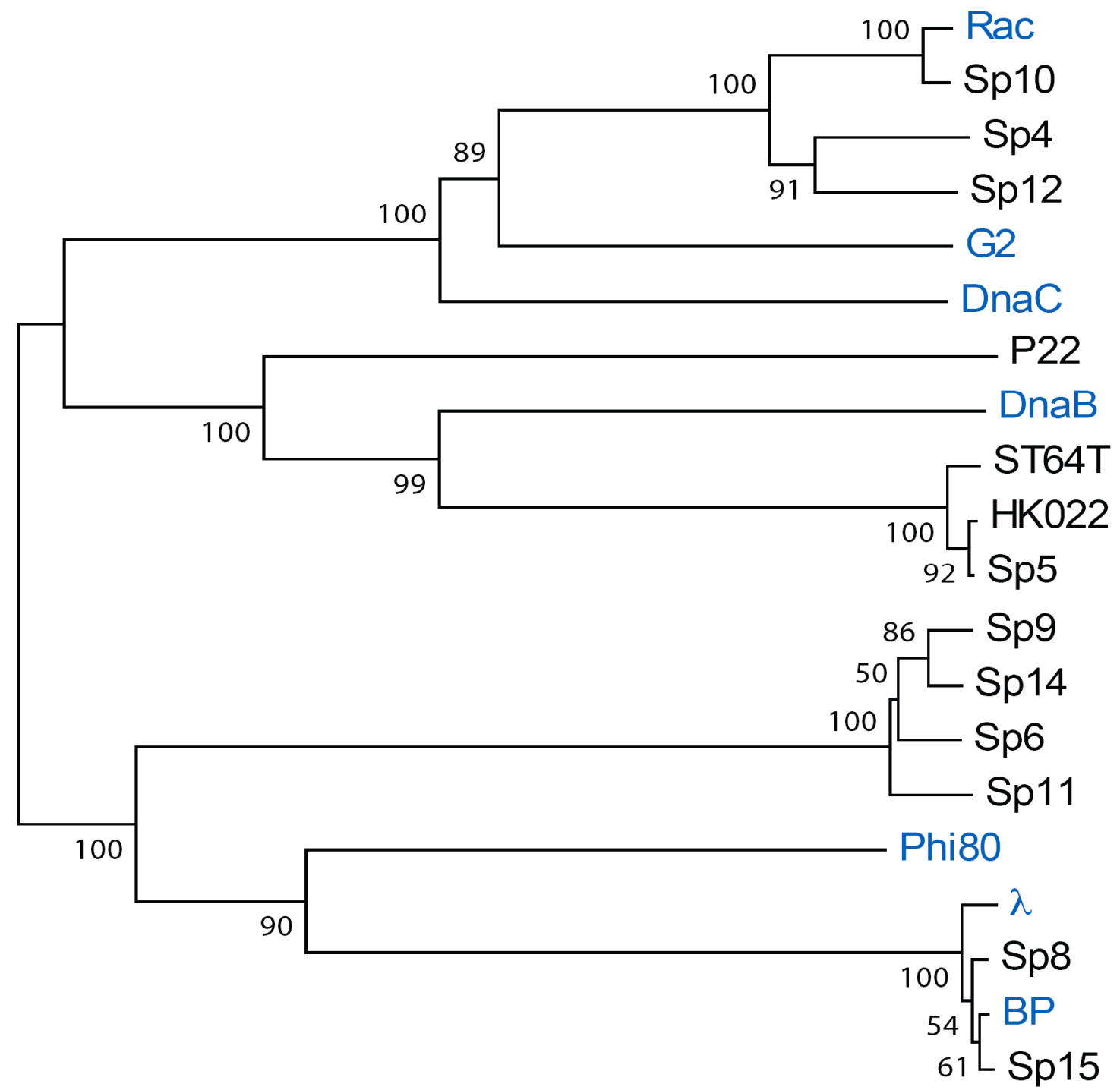

Supplement: Figure S3 — Replication elongation proteins of O157 Sakai lambdoid prophages. Amino acid sequences of replication elongation proteins of lambdoid Sps were compared with those of reference proteins from other lambda phage family members. A neighbor-joining (N-J) tree constructed based on the sequence comparisons is shown. Reference sequences are from phage lambda (λ), enterobacteria phage P22 (P22), phage BP4795 (BP), bacteriophage phi80 (phi80), K-12 cryptic prophage Rac (Rac), enterobacteria phage HK022 (HK022), Salmonella phage Gifsy-2 (G2), and enterobacteria phage ST64T (ST64T), E. coli DnaB (DnaB_Ec), and E. coli DnaC (DnaC_Ec). (0.07 MB PDF) [file ppat.1000408.s003.pdf]

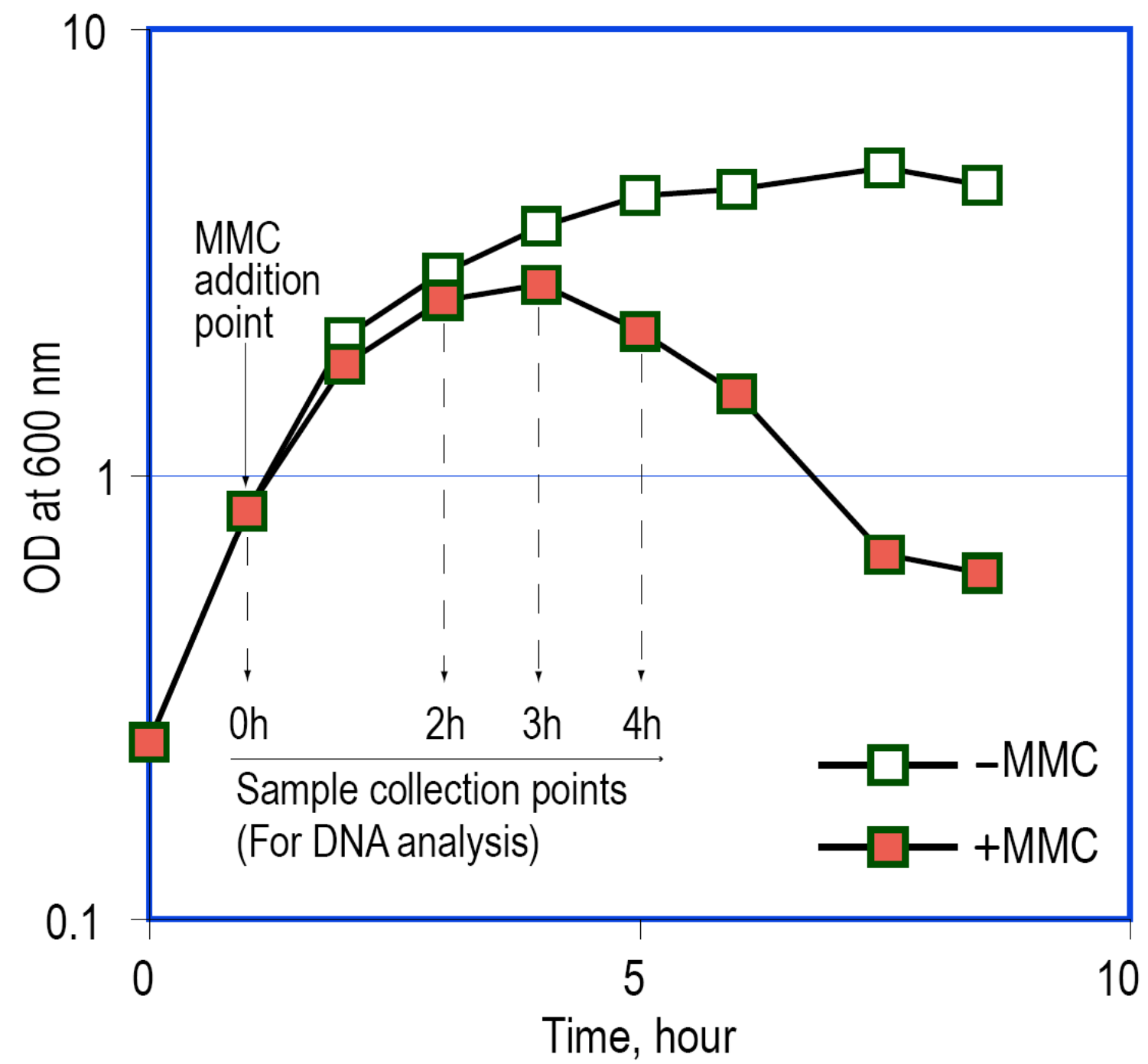

Supplement: Figure S4 — Cell lysis of MMC-treated O157 Sakai cells. Growth of O157 Sakai cells in the absence (−MMC) or presence (+MMC) of MMC was monitored by measuring the OD600 of each culture. Time points of sample collection for microarray analysis (Figure 2) are indicated. MMC was added to the culture at the 0-h point. (0.04 MB PDF) [file ppat.1000408.s004.pdf]

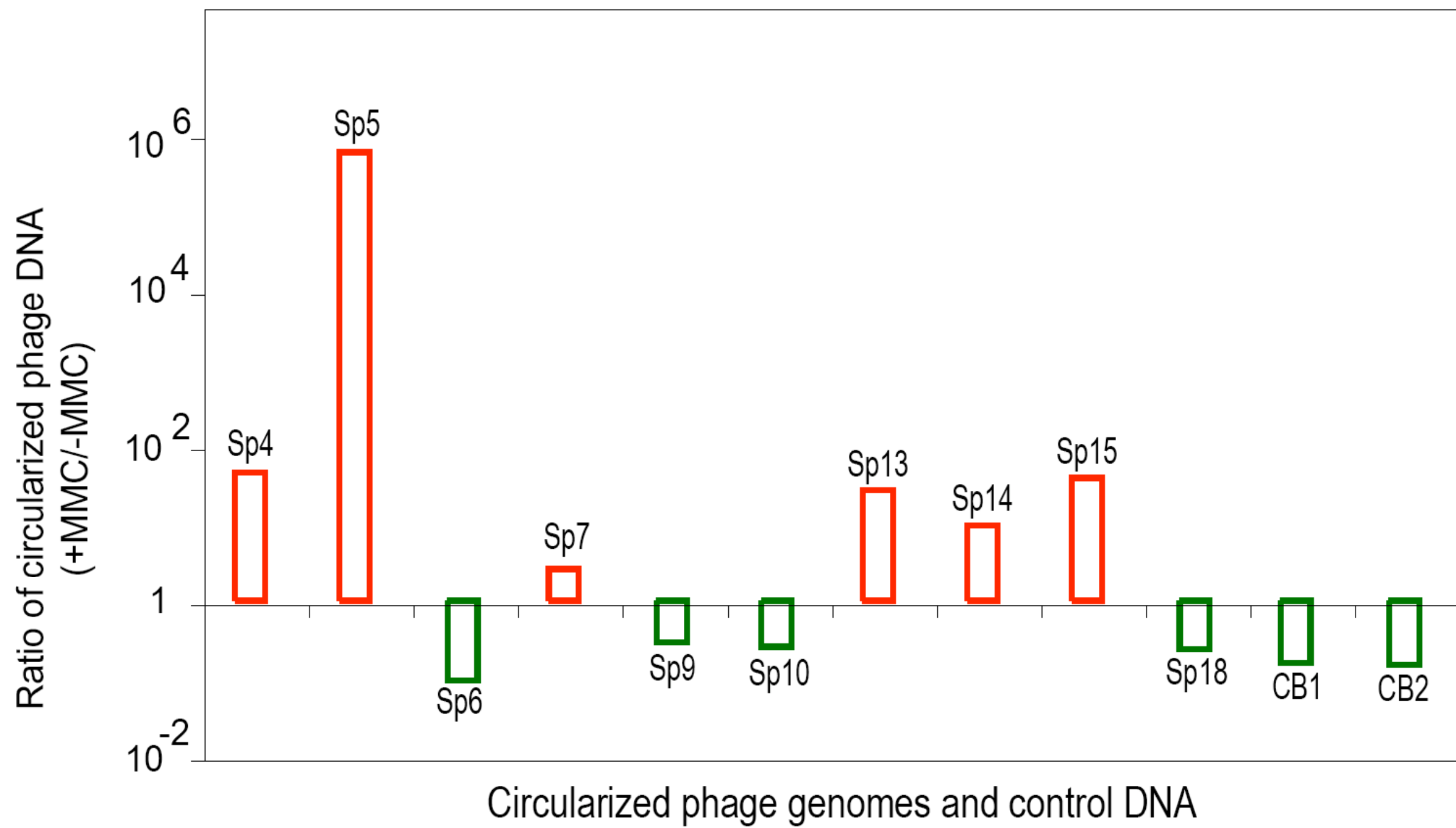

Supplement: Figure S6 — Ratios of MMC-induced and spontaneously induced prophage genomes. Ratios of circularized prophage genomes in MMC-treated O157 Sakai cells to those in untreated cells are shown for each of the nine Sps that were found to be excised and circularized in O157 Sakai cells. As controls, amounts of the Sp18 DNA and chromosomal DNA from two regions (chromosomal backbone, CB1 and CB2) were measured. Note that Sp18 is not inducible by MMC treatment, and its genome is not circularized, similar to results previously reported for the prototype Mu phage. (0.04 MB PDF) [file ppat.1000408.s006.pdf]

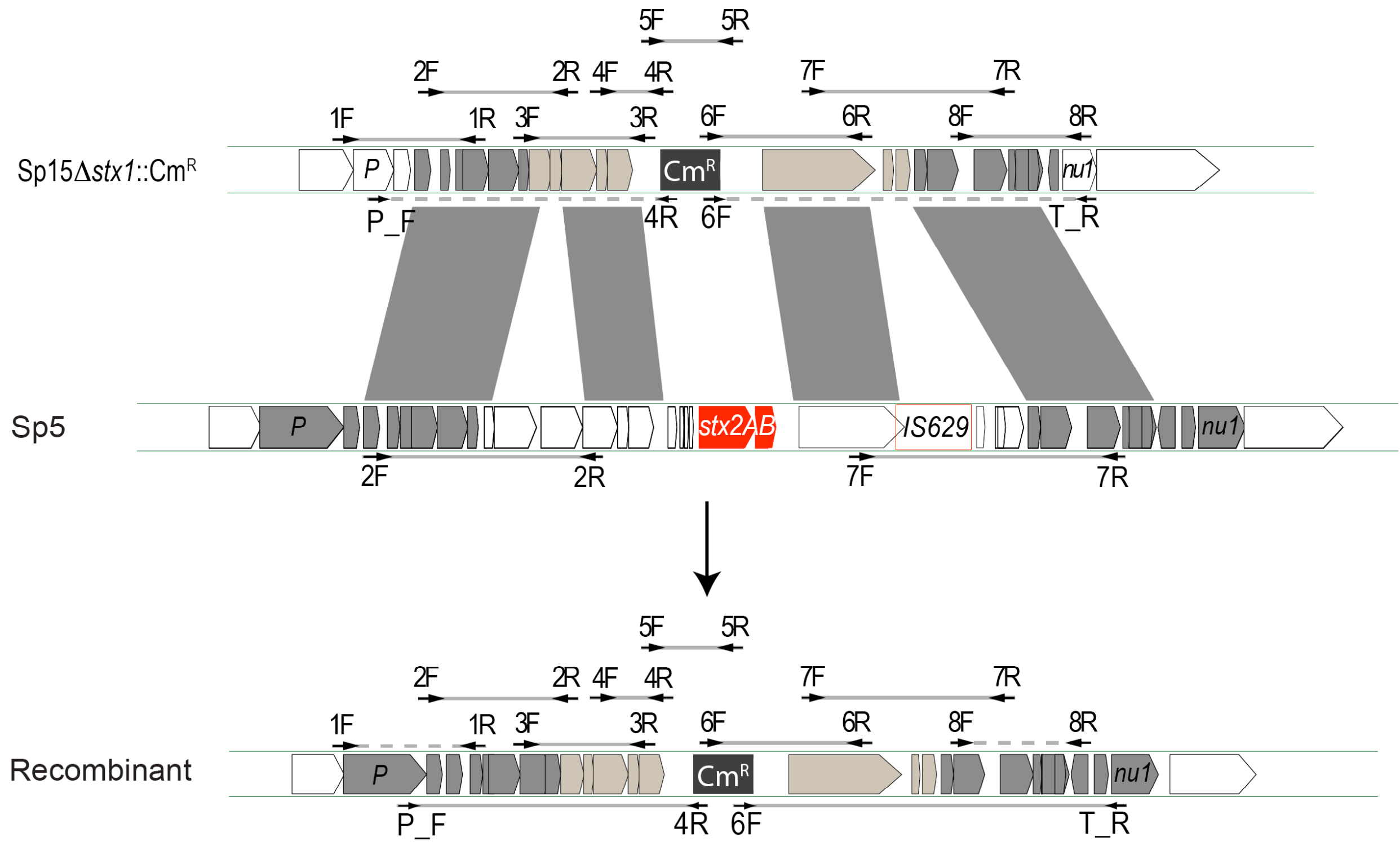

Supplement: Figure S7 — Sequence homology between Sp5 and Sp15 genomes and PCR scanning analysis of Sp5/Sp15 recombinant phages. Gene organizations of the stx1 - and stx2-flanking regions of Sp15 and Sp5 are shown. The stx1 gene of Sp15 has been replaced by the CmR cassette. Highly homologous regions (>90% nucleotide sequence identity) that probably mediated the recombination between the Sp5 and Sp15 genomes are indicated by gray shading. PCR primer positions used for PCR scanning analysis of Sp5/Sp15 recombinant phages (RP) are indicated by arrows. The results of PCR amplification shown in Figure 6 of the main text are indicated by solid (amplified) or dotted (not amplified) lines between the primer pair. (0.13 MB PDF) [file ppat.1000408.s007.pdf]
