# Supplementary material for: The Defective Prophage Pool of Escherichia coli O157: Prophage–Prophage Interactions Potentiate Horizontal Transfer of Virulence Determinants
Source: PLoS Pathog. 2009 May 1;5(5):e1000408. doi: 10.1371/journal.ppat.1000408 (PMC2669165; doi:10.1371/journal.ppat.1000408)
Supplement: Figure S2 — Schematic presentation of the domain structures of repressors and antirepressors of O157 Sps and their corresponding prototype phages. Functional motifs identified in the predicted repressors and antirepressors of Sps are shown. Motifs in repressors and antirepressors of prototype phages and their relatives are also shown. (0.07 MB PDF) [file ppat.1000408.s002.pdf]

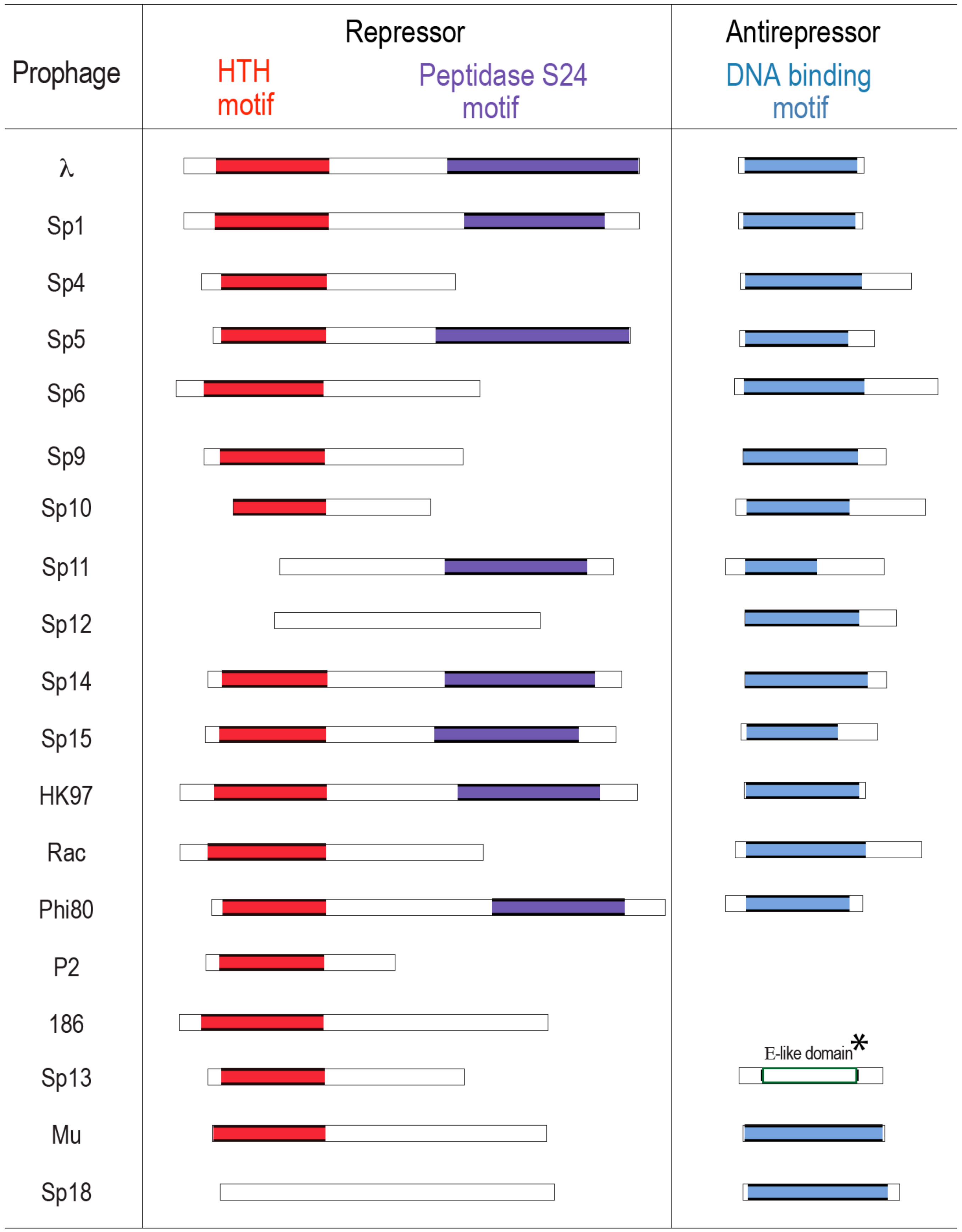

* The domain is found in the P4 ε gene product (E) that does not have a DNA binding motif but can bind to repressor C of P2-like prophages to derepress the helper P2 genome [30].
