# Supplementary material for: The Defective Prophage Pool of Escherichia coli O157: Prophage–Prophage Interactions Potentiate Horizontal Transfer of Virulence Determinants
Source: PLoS Pathog. 2009 May 1;5(5):e1000408. doi: 10.1371/journal.ppat.1000408 (PMC2669165; doi:10.1371/journal.ppat.1000408)
Supplement: Figure S5 — Attachment site (attP) sequences of Sakai prophages. DNA sequences of attachment sites (attP) of the nine Sps that were found to be excised and circularized in O157 Sakai cells are shown. Right (R) and left (L) junctions of prophage regions on the O157 Sakai chromosome are aligned with the attP sequence determined in this study (P). Core attachment site sequences are indicated in blue. Nucleotide positions on the O157 Sakai chromosome are also indicated for right and left junctions. (0.10 MB PDF) [file ppat.1000408.s005.pdf]

Sp4	1161086	ttatcttatctggcggaagcgcagagattcgaactctggaaccctttcgggtcgccggttttcaagaccgaagaa	1161160	L
		cttgattatttggcggaagcgcagagattcgaactctggaaccctttcgggtcgccggttttcaagaccgaagaa		P
	1210877	cttgattatttggcggaagcgcagagattcgaactctggaaccctttcgggtcgccggttttcaagaccggtgcc	1210951	R
Sp5	1246148	ccatcgtttcaacatgtctaagg	1246170	L
		tcatggtttcaacatgtctaagg		P
	1308856	tcatggtttcaatatgtccgtac	1308878	R
Sp6	1541606	ttccaccttttattcataagg	1541627	L
		tgttgccttcttattcataagg		P
	1590029	tgttgccttcttattcagcaga	1590050	R
Sp7	1594706	agtatggacatattttatca	1594725	L
		taaaaggacatattttatca		P
	1610169	taaaaggacatatttattca	1610188	R
Sp9	1757521	catgtcagtgtggtacatggatatcgataccacggcta	1757558	L
		tctaccagtgtggtacatggatatcgataccacggcta		P
	1815696	tctaccagtgtggtacatggatatcgataccaccgcca	1815733	R
Sp10	1921506	tgtaatttgttcaggttgtattgttctttctttgtaatttgttgattttcttgc	1921556	L
		ctgacttgttcaggttgtattgttctttctttgtaatttgttgattttcttgc		P
	1972619	ctgacttgttcaggttgtattgttctttcttgctaatttcttgattttgcgac	1972617	R
Sp13	2593037	gcacaaaaaaaccacccgaaggtggtttcacgacactgcttattgctttgattttattcttatctttcccatggtacccggagcgggacttgaacccgcacagcgcgaacgccgagggattttaaaaacta	2593167	L
		atacgaaaaaaccacccgaaggtggtttcacgacactgcttattgctttgattttattcttagctttcccatggtacccggagcgggacttgaacccgcacagcgcgaacgccgagggattttaaaaacta		P
	2614157	atacgaaaaaaccacccgaaggtggtttcacgacactgcttattgctttgattttattcttagctttcccatggtacccggagcgggacttgaacccgcacagcgcgaacgccgagggattttaaatccct	2614287	R
Sp14	2668143	taaagtggcggagagagggggatttgaacccccggtagagttgcccctactccggttttcgagacctatgc	2668213	L
		aattttggcggagagagggggatttgaacccccggtggagttgcccccactccggttttcgagacctatgc		P
	2712172	aattttggcggagagagggggatttgaacccccggtggagttgcccccactccggttttcgagaccggtcc	271224	R
Sp15	2896041	attaaccctgtcacgttacgcgcgtgacgca	2896071	R
		ttaatccctgtcacgttacgcgcgtgacgca		P
	2943921	ttaatccctgtcacgttacgcgcgtggcaga	2943951	L
