# Supplementary material for: The Defective Prophage Pool of Escherichia coli O157: Prophage–Prophage Interactions Potentiate Horizontal Transfer of Virulence Determinants
Source: PLoS Pathog. 2009 May 1;5(5):e1000408. doi: 10.1371/journal.ppat.1000408 (PMC2669165; doi:10.1371/journal.ppat.1000408)
Supplement: Figure S8 — Sequence alignments of the cos sequences and the Nu1 subunit proteins of Sp4, Sp14, and phage lambda. (A) DNA sequences of the cos sites of Sp4 and Sp14 are identical. (B) Amino acid sequences of the Nu1 proteins of Sp4 and Sp14 are also identical, but the corresponding sequence in Sp4 has been disrupted by an ISEc8-insertion. (0.10 MB PDF) [file ppat.1000408.s008.pdf]

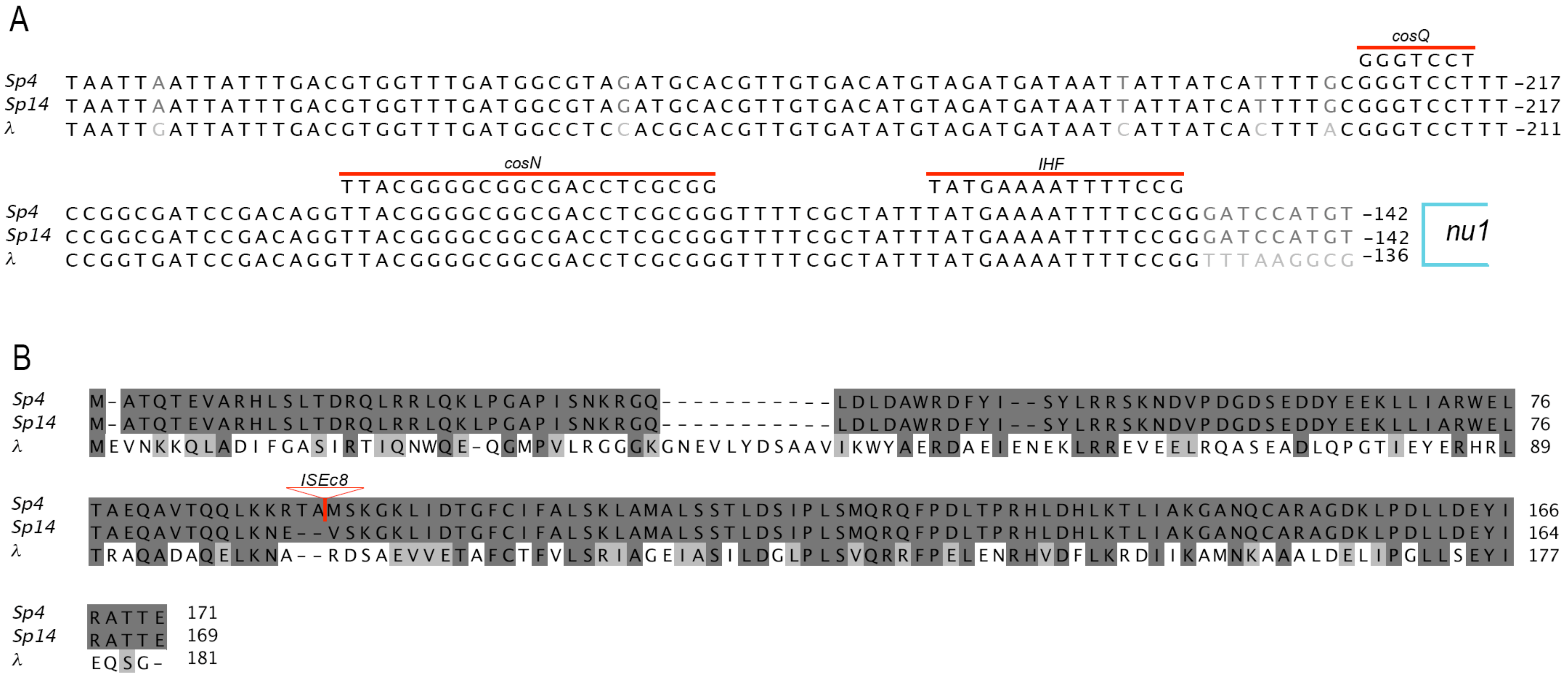

A
cosQ
GGGTCCT
Sp4 TAATTAATTATTTGACGTGGTTTGATGGCGTAGATGCACGTTGTGACATGTAGATGATAATTATTATCATTTTGCGGGTCCTTT -217
Sp14 TAATTAATTATTTGACGTGGTTTGATGGCGTAGATGCACGTTGTGACATGTAGATGATAATTATTATCATTTTGCGGGTCCTTT -217
λ TAATTGATTATTTGACGTGGTTTGATGGCCTCCACGCACGTTGTGATATGTAGATGATAATCATTATCACTTTACGGGTCCTTT -211
cosN
TTACGGGGCGGCGACCTCGCGG
IHF
TATGAAAATTTTCCG
Sp4 CCGGCGATCCGACAGGTTACGGGGCGGCGACCTCGCGGGTTTTCGCTATTTATGAAAATTTTCCGGGATCCATGT -142
Sp14 CCGGCGATCCGACAGGTTACGGGGCGGCGACCTCGCGGGTTTTCGCTATTTATGAAAATTTTCCGGGATCCATGT -142
λ CCGGTGATCCGACAGGTTACGGGGCGGCGACCTCGCGGGTTTTCGCTATTTATGAAAATTTTCCGGTTTAAGGCG -136
nu1
B
Sp4 M-ATQTEVARHLSLTDRQLRRLQKLPGAPISNKRGQ------------LDLDAWRDFYI--SYLRRSKNDVPDGDSEDDYEEKLLIARWEL 76
Sp14 M-ATQTEVARHLSLTDRQLRRLQKLPGAPISNKRGQ------------LDLDAWRDFYI--SYLRRSKNDVPDGDSEDDYEEKLLIARWEL 76
λ MEVNKKQLADIFGASIRTIQNWQE-QGMPVLRGGGKGNEVLYDSAAVIKWYAERDAEIENEKLRREVEELRQASEADLQPGTIEYERHRL 89
ISEc8
Sp4 TAEQAVTQQLKKRTAMSKGKLIDTGFCIFALSKLAMALSSTLDSIPLSMQRQFPDLTPRHLDHLKTLIAKGANQCARAGDKLPDLLDEYI 166
Sp14 TAEQAVTQQLKNE--VSKGKLIDTGFCIFALSKLAMALSSTLDSIPLSMQRQFPDLTPRHLDHLKTLIAKGANQCARAGDKLPDLLDEYI 164
λ TRAQADAQELKNA--RDSAEVVETAFCTFVLSRIAGEIASILDGLPLSVQRRFPELENRHVDFLKRDIIKAMNKAAALDELIPGLLSEYI 177
Sp4 RATTE 171
Sp14 RATTE 169
λ EQSG- 181
