# Supplementary material for: The Defective Prophage Pool of Escherichia coli O157: Prophage–Prophage Interactions Potentiate Horizontal Transfer of Virulence Determinants
Source: PLoS Pathog. 2009 May 1;5(5):e1000408. doi: 10.1371/journal.ppat.1000408 (PMC2669165; doi:10.1371/journal.ppat.1000408)
Supplement: Table S1 — Primers and TaqMan probes for qualitative PCR and real-time qPCR. (0.08 MB DOC) [file ppat.1000408.s009.doc]

Table S1: Primers and TaqMan probes for qualitative PCR and real-time qPCR

| Prophages | Sequences | |
| --- | --- | --- |
| *attP* detection primers | |  |
|  | Forward | Reverse |
| Sp1 | GATGGACTGTTCCGGTACTTTATC | GTCAGTGTTACAGGAAATGGGAG |
| Sp1-2 | GAGTAGTATGAACAGTTTTCCCCC | CGGAAGAGAGTAAATCAAACCG |
| Sp2 | GAGTAGTATGAACAGTTTTCCCCC | AACCTCACTAGGGTTCTTTCCTTC |
| Sp3 | GATGACAATACAGTGAGTCTGGGA | TGTCTGCAAGACTCTATGAGAAGC |
| Sp4 | GAATATCAGGGACAAGGTGAGTGT | GTGGTTAGCAAGTAGACAATCCAG |
| Sp5 | CCTTTGTACGGATGTAACTATGCC | GATCCCGTAAAGCGTATCAGTC |
| Sp6 | TTGCGAATACTTTCTCCAGTCC | GTCCATATCTAATCCATAGTCGGC |
| Sp7 | ACTGGTTGATACTTTCGACCTCCT | CTCAGTTTGTTAAGTGCTCTGCTC |
| Sp8 | GACAGCCGTAATGATATTCCTGAG | ACTACCTCTCAAGCAAACAGAGGA |
| Sp9 | AGGATGCGACTATTGTGGTAGAAC | GAGCAGAGATCTTTATCAGAGCGA |
| Sp10 | GGGCGCCAGATTGATTAGTTATAG | GACGATACTGGATATGTTCGGTC |
| Sp11 | ACGAGACTGAGCAAAGAGCATATC | GTCATAACGTTTACTGACACGTCG |
| Sp12 | CAATTTCCTCTCGAGTAACAGACC | AGGTAATACACCGTAAGAGCTGGA |
| Sp13 | GTTTAACAGAGGTCCTGGTTCACT | GGCAATTAACTCATGTGCTACGTC |
| Sp14 | GGCTTTAACGAAATGTGGGTAG | CAGAAGAGAGGTTAAATACCGTGG |
| Sp15 | TCACTACAAGGTTGACTCCATCAG | GGCTTTTACGATCACCTCTACATC |
| Sp16 | AGATCGTTAGAGAGATGTTGGAGC | ATACAGGGACATAAAGGTACAGGC |
| Sp17 | TCACCTCTCTCGTATGTTCAACTG | TCCAGGTATCTTTCAGAGTCTCCT |
| Quantitation primers | | |
|  | Forward | Reverse |
| Sp4 | CGCTCATGACCGATAATCTCAT | GATTAACTTTTCAGCAATTTCACG |
| Sp5 | CTCTTGGACGATCTTCGGTAA | CGTGACTCAAGTTGCCATGT |
| Sp6 | TTTATGGATAGAGGCATGCTGTTTAC | AATGCGTGGCCTAGTTTTGG |
| Sp7 | TTTTTGCAGTGTGGGTTGTTTAATTA | ACATTATGAAACGGCAAAAAACACTAA |
| Sp9 | CAAAATCAATCAGTTAAAAACACGA | GGATCCAGTCGTACCGAGTC |
| Sp10 | TTCGTCAGATAGTTGCGGTTTTTA | GAATGCGCGGTTATCATAGTTG |
| Sp13 | CCATTGCACAAAATACAAATACG | TGGCGTTGTATAGAGCCATTT |
| Sp14 | CAACACTTGATGTCCCGTTACC | ATTGATTTTATTAACCCATAGCATAG |
| Sp15 | TCGACATGGTAAACGAATCATCTATC | CCATAAGAAAGCAGGGTAAAATAAAAAC |
| Sp18^a^ | AAGGGGAAATATAAAGGCGATAAC | ATGACGTTAATACAGCCCATCAG |
| CB1 | AGCGGTAAGTCGGCTTCATC | TCAGGTGGCAAAGGCTATCG |
| CB2 | GCTCTCTGCGTTTAGACTTAATTTCA | TGTCTCGGCAGTATGATAATTGTCTT |
| TaqMan Probes | | |
| Sp4 | TTTCGGGTCGCCGGTTTTCAA | |
| Sp5 | CTTCATGGTTTCAACATGTCTAAGGAT | |
| Sp6 | TATTCATAAGGCGGGACACTTTCAGGATTTT | |
| Sp7 | TGGGTTGCCTTGCTGTGGTGATAAA | |
| Sp9 | TGTGGTACATGGATATCGATACCAC | |
| Sp10 | TGACTTGTTCAGGTTGTATTGTTCTTTCTTTGTAATTTGT | |
| Sp13 | CGCCGAGGGATTTTAAAAAC | |
| Sp14 | AAGTGATTGAATTTTGGCGG | |
| Sp15 | CGCGTAACGTGACAGGGATTAAGGGTAAA | |
| Sp18^a^ | TGGAAACGCAGACAGTTCAG | |
| CB1^b^ | CCTGCAATTCAATCAGAGTCGTCA | |
| CB2^b^ | TCTTGTTCCTTGTATTCTCTTTTAAC | |

^a^ An internal region was used to detect Sp18 DNA.

^b^ Two chromosomal backbone regions (CB1 and CB2) were used as controls.
