# Supplementary material for: The Defective Prophage Pool of Escherichia coli O157: Prophage–Prophage Interactions Potentiate Horizontal Transfer of Virulence Determinants
Source: PLoS Pathog. 2009 May 1;5(5):e1000408. doi: 10.1371/journal.ppat.1000408 (PMC2669165; doi:10.1371/journal.ppat.1000408)
Supplement: Table S2 — Primers used to construct CmR-marked prophages and verify the insertion of the CmR gene cassette. (0.16 MB DOC) [file ppat.1000408.s010.doc]

Table S2. Primers used to construct Cm^R^-marked prophages and to verify the insertion of the Cm^R^ gene cassette.

| Primer  Name | Prophage | Primer Sequence | Amplicon Size (bp) | |
| --- | --- | --- | --- | --- |
| Gene replacement primers^b^ | | | | |
| Δ1074_F | Sp4 | TAGCTAGATTGATAAAGTGATTACATATTTTCTGttatatgctga  aatgaataatgtgtaggctggagctgcttc | 1,143 | |
| Δ1074_R |  | CTTAAGCATCCTTTAATGCTCATGGGGAGAACGCatgactaaac  atatggacattatgggaattagccatggtcc |  |  |
| Δ*stx2*_F | Sp5 | ATGAAGTGTATATTATTTAAATGGGTACTGTGCCTGTTACTGGGT  TTTTCTTCGGTATCCTATTCCgtgtaggctggagctgcttc | 1,190 | |
| Δ*stx2*_R |  | TCAGTCATTATTAAACTGCACTTCAGCAAATCCGGAGCCTGATTC  ACAGGTatgggaattagccatggtcc |  |  |
| Δ*espN*_F | Sp6 | TACCAGAGGAGGCAGGACATAGATTGTAAAAAAActatttcgat  gcatttaccatatgggaattagccatggtcc | 1,143 | |
| Δ*espN*_R |  | TGAATAGTGCGTTATAAGAGGAAGTGGAATTTTAatgaaaataa  caaactatatagtgtaggctggagctgcttc |  |  |
| Δ1585_F | Sp7 | GGTCATTTGCCACCTCAATTTATATATATAGAAACatgtgcggg  ttatgcgggttaatgggaattagccatggtcc | 1,144 | |
| Δ1585_R |  | TTTACGTGCTGATGTTCCAGCCTGATGATGAAGAttaatcccct  gtgtgaggaaagtgtaggctggagctgcttc |  |  |
| Δ1772_F | Sp9 | ATGAGGAACATAATGGCAGGTTTTTTAATATTCCTGTCTTCTGC  TGCTTATGCTgtgtaggctggagctgcttc | 1141 | |
| Δ1772_R |  | TCAAGTGCCTTTCCTGGTCCAGCCATATTTTTTGAATGCAGGCG  CCGCTTCATCatgggaattagccatggtcc |  |  |
| Δ1953_F | Sp10 | AAATGAACGGGATAACTATTATAAATACTATAGGttataccctaa  cttccaaagaatgggaattagccatggtcc | 1,143 | |
| Δ1953_R |  | ATACATAGAAACTAATAAGAGACGTTGCAACTATatgaatgtaat  agatttgtttgtgtaggctggagctgcttc |  |  |
| Δ2638_F | Sp13 | AAGGAGATTATCGTGCTATCTTACTTAATGGCAATTCACTTTGTT  GTGTAGGCTGGAGCTGCTTC | 1,123 | |
| Δ2638_R |  | AATATCGATGGCTTTATGTACTCTATTTATACAATACAACACCAC  TCTTATGGGAATTAGCCATGGTCC |  |  |
| Δ*stx1*_F | Sp15 | TCAACGAAAAATAACTTCGCTGAATCCCCCTCCATTATGACAGGC  ATTAGTgtgtaggctggagctgcttc | 1,175 | |
| Δ*stx1*_R |  | ATGAAAATAATTATTTTTAGAGTGCTAACTTTTTTCTTTGTTATCTT  TTCAatgggaattagccatggtcc |  |  |
| Primers to verify replacement | | | Before^b^ | After^b^ |
| V1074_F | Sp4 | GATGGGAAAAGAGCAGATACATAGA | 1,280 | 1,581 |
| V1074_R |  | CTAAGCAAGCGATGTAAACGATAAT |  |  |
| V*stx2*_F | Sp5 | TTTATTTACCAGGCTCGCTTTTG | 1,581 | 1,490 |
| V*stx2*_R |  | GCCTTGGTATATGCCTAATCTCTG |  |  |
| V*espN*_F | Sp6 | GATAGAACTCGTTGTGATTCGATTT | 3,889 | 1,562 |
| V*espN*_R |  | AGATGTAGCATCGTTTTTACCAGAG |  |  |
| V1585_F | Sp7 | GAATTTAAACAGATGGTTGAACAGG | 778 | 1,508 |
| V1585_R |  | ATCTAACGCTTTATATTTCGGCTCT |  |  |
| V1772_F | Sp9 | TATTCCTGTCTTCTGCTGCTTATG | 1,226 | 1,608 |
| V1772_R |  | CTTTTATCTATCTGCCGTTGGTGT |  |  |
| V1953_F | Sp10 | ATTAAATTTATGCGATCCGGTATTT | 1,461 | 1,384 |
| V1953_R |  | CCTTTCTAACTGGCAATTTTATTCA |  |  |
| V2638_F | Sp13 | CATGTATGTCCTGACCGGAAC | 1037 | 1557 |
| V2638_R |  | GATACAACAGTAGCAGCGGATG |  |  |
| V*stx1*_F | Sp15 | AACGAAAAATAACTTCGCTGAATC | 1,581 | 1,489 |
| V*stx1*_R |  | TGCACATGGCACTATTAGTTTTCT |  |  |

^a^ Underlined regions in forward and reverse primers correspond, respectively, to priming sites 1 and 2 in the template plasmid pKD3.

^b^ Expected sizes of amplicons before and after gene replacement are shown.
