# Supplementary material for: The Defective Prophage Pool of Escherichia coli O157: Prophage–Prophage Interactions Potentiate Horizontal Transfer of Virulence Determinants
Source: PLoS Pathog. 2009 May 1;5(5):e1000408. doi: 10.1371/journal.ppat.1000408 (PMC2669165; doi:10.1371/journal.ppat.1000408)
Supplement: Table S3 — Bacterial strains and plasmids used in this study. (0.06 MB DOC) [file ppat.1000408.s011.doc]

Table S3. Bacterial strains and plasmids used in this study

| Strains, Plasmids | Genetic features | References  or sources |
| --- | --- | --- |
| Bacterial strains | | |
| Sakai | *E. coli* O157:H7 strain Sakai | [Watanabe *el at*., 1996]^a^ |
| AH31 | O157 Sakai derivative; Δ*ECs1077*::*cat* in Sp4 | This study |
| AH32 | O157 Sakai derivative; Δ*stx2AB*::*cat* in Sp5 | This study |
| AH33 | O157 Sakai derivative; Δ*espN*::*cat* in Sp6 | This study |
| AH34 | O157 Sakai derivative; Δ*paa*::*cat* in Sp9 | This study |
| AH36 | O157 Sakai derivative; Δ*ECs1953*::*cat* in Sp10 | This study |
| AH20 | O157 Sakai derivative; Δ*ECs2638*::*cat* in Sp13 | This study |
| AH38 | O157 Sakai derivative; Δ*stx1AB*::*cat* in Sp15 | This study |
| AH846 | O157 Sakai strain carrying pKD46 | This study |
| MG1655 | *E. coli* K-12 derivative | [Blattner *et al.,* 1997]^b^ |
| MC1061 | *E. coli* K-12 derivative | [Wertman *et al.,* 1986]^c^ |
| Plasmids | | |
| pKD3 | Template plasmid with the Cm^R^ gene cassette | [Datsenko *et al*., 2000]^d^ |
| pKD46 | Red recombinase expression vector | [Datsenko *et al*., 2000]^d^ |

^a^[Watanabe H](http://www.ncbi.nlm.nih.gov/sites/entrez?Db=pubmed&Cmd=Search&Term="Watanabe H"%5BAuthor%5D&itool=EntrezSystem2.PEntrez.Pubmed.Pubmed_ResultsPanel.Pubmed_RVAbstractPlus), [Wada A](http://www.ncbi.nlm.nih.gov/sites/entrez?Db=pubmed&Cmd=Search&Term="Wada A"%5BAuthor%5D&itool=EntrezSystem2.PEntrez.Pubmed.Pubmed_ResultsPanel.Pubmed_RVAbstractPlus), [Inagaki Y](http://www.ncbi.nlm.nih.gov/sites/entrez?Db=pubmed&Cmd=Search&Term="Inagaki Y"%5BAuthor%5D&itool=EntrezSystem2.PEntrez.Pubmed.Pubmed_ResultsPanel.Pubmed_RVAbstractPlus), [Itoh K](http://www.ncbi.nlm.nih.gov/sites/entrez?Db=pubmed&Cmd=Search&Term="Itoh K"%5BAuthor%5D&itool=EntrezSystem2.PEntrez.Pubmed.Pubmed_ResultsPanel.Pubmed_RVAbstractPlus), and [Tamura K](http://www.ncbi.nlm.nih.gov/sites/entrez?Db=pubmed&Cmd=Search&Term="Tamura K"%5BAuthor%5D&itool=EntrezSystem2.PEntrez.Pubmed.Pubmed_ResultsPanel.Pubmed_RVAbstractPlus) (1996) [Lancet](javascript:AL_get(this, 'jour', 'Lancet.');) 348: 831-832. ^b^Blattner FR, Plunkett G 3rd, Bloch CA, Perna NT, Burland V, et al. (1997) Science 277: 1453-1474. **^c^**[Wertman KF](http://www.ncbi.nlm.nih.gov/sites/entrez?Db=pubmed&Cmd=Search&Term="Wertman KF"%5BAuthor%5D&itool=EntrezSystem2.PEntrez.Pubmed.Pubmed_ResultsPanel.Pubmed_RVAbstractPlus), [Wyman AR](http://www.ncbi.nlm.nih.gov/sites/entrez?Db=pubmed&Cmd=Search&Term="Wyman AR"%5BAuthor%5D&itool=EntrezSystem2.PEntrez.Pubmed.Pubmed_ResultsPanel.Pubmed_RVAbstractPlus), and [Botstein D](http://www.ncbi.nlm.nih.gov/sites/entrez?Db=pubmed&Cmd=Search&Term="Botstein D"%5BAuthor%5D&itool=EntrezSystem2.PEntrez.Pubmed.Pubmed_ResultsPanel.Pubmed_RVAbstractPlus) (1986) Gene 49: 253-262. ^d^Datsenko KA and Wanner BL (2000) [Proc Natl Acad Sci U S A](javascript:AL_get(this, 'jour', 'Proc Natl Acad Sci U S A.');) 97: 6640–6645.
