# Supplementary material for: The Defective Prophage Pool of Escherichia coli O157: Prophage–Prophage Interactions Potentiate Horizontal Transfer of Virulence Determinants
Source: PLoS Pathog. 2009 May 1;5(5):e1000408. doi: 10.1371/journal.ppat.1000408 (PMC2669165; doi:10.1371/journal.ppat.1000408)
Supplement: Table S4 — Primers used to examine integration and lysogenization of Sakai prophages in K-12 strains. (0.08 MB DOC) [file ppat.1000408.s012.doc]

Table S4. Primers used to examine integration and lysogenization of Sps in K-12 strains.

| Primer  Name | Sequences | Amplicon  Size (bp) |
| --- | --- | --- |
| Sp5 | | |
| LbF | ataagtttgatagcgtgttgaatgaa | 1,149 |
| LbR | GATCCCGTAAAGCGTATCAGTC |
| Pi5F | GCGAAAATACCACAAACCTTTTC | 1,435 |
| Pi5R | ACGTAATAATCAACCAGCAAGTCA |
| RbF | CCTTTGTACGGATGTAACTATGCC | 724 |
| RbR | ctccgtctgggtatagacatcatatt |
| Sp6 | | |
| LbF | tactcggtccagttgtagaaatacag | 1,342 |
| LbR | GTCCATATCTAATCCATAGTCGGC |
| Pi6F | CCTAATAGGTGATGGTTTGTGG | 1,492 |
| Pi6R | CATTAGCCATACTCACCTCATCTG |
| RbF | TTGCGAATACTTTCTCCAGTCC | 1,078 |
| RbR | gatgaagaacacaagtaagttccaga |
| Sp10 | | |
| LbF | tttcaggtatacaggaatgctaacag | 1,074 |
| LbR | gtcagaagaaacctGACGATACTG |
| Pi10F | CCAACCTGTGAGATGTGTCTTATC | 1,740 |
| Pi10R | AAAGCCTCAAACGACTTTAGCAAC |
| RbF | GGGCGCCAGATTGATTAGTTATAG | 1,085 |
| RbR | tataaaatcaacctgttggatgaaga |
| Sp15 | | |
| LbF | ttatcgagcaatatacgcataaacag | 1,235 |
| LbR | GGCTTTTACGATCACCTCTACATC |
| Pi15F | AGTCGTAATCTTCACCACTTCGAT | 1,451 |
| Pi15R | GGTGTCATTAACCCTATCCAGAAC |
| RbF | TCACTACAAGGTTGACTCCATCAG | 1,161 |
| RbR | aaaataaaggtataacctgcgagcat |
