# Supplementary material for: The Defective Prophage Pool of Escherichia coli O157: Prophage–Prophage Interactions Potentiate Horizontal Transfer of Virulence Determinants
Source: PLoS Pathog. 2009 May 1;5(5):e1000408. doi: 10.1371/journal.ppat.1000408 (PMC2669165; doi:10.1371/journal.ppat.1000408)
Supplement: Table S5 — Primers used to analyze the Sp15/Sp5 recombinant phage. (0.05 MB DOC) [file ppat.1000408.s013.doc]

**Table S5. Primers used to analyze the Sp15/Sp5 recombinant phage.**

| Primer names | Sequences | Amplicon sizes  (bp) |
| --- | --- | --- |
| 1F | GTAATGGGTGTGTGATTAATGCT | 3,881 |
| 1R | GAACATGATTTCGATAAGCTCAG |
| 2F | CAGGAATCAAAGAAAAGAATTT | 2,394 |
| 2R | TGCTAACTGAGTTCCGTGTTC |
| 3F | CGAAGTATCACCGACATCATC | 2,296 |
| 3R | ATCTCATGCGACTACTTGACG |
| 4F | tatggtgctcaaggagtattgtgta | 1,330 |
| 4R | GATCTTCCGTCACAGGTAGG |
| 5F | TGTCAACGGTGTTCTTATGGT | 1,148 |
| 5R | GATCTTCCGTCACAGGTAGG |
| 6F | TTATACGCAAGGCGACAAGG | 2,646 |
| 6R | TGTCTGTCACATGCAGTTTATCC |
| 7F | GAATATCCATTTCGTACCGTTCA | 3,292 |
| 7R | TCACCAGCGTTATGAGTTTATCT |
| 8F | GAAAAATGAGGTTTGCTTACAT | 2,304 |
| 8R | TGTACTTCATTCAGTTGTGATGC |
| P_F | AACTAATCCTTCGTCTCAATCGTC | - |
| 4R | GATCTTCCGTCACAGGTAGG |
| 6F | TTATACGCAAGGCGACAAGG | - |
| T_R | GTCCAGTAATGTTATTCCCGTTTC |
